# In vitro culture at 39 °C during hepatic maturation of human ES cells facilitates hepatocyte-like cell functions

**DOI:** 10.1038/s41598-022-09119-7

**Published:** 2022-03-25

**Authors:** Satoshi Imamura, Koki Yoshimoto, Shiho Terada, Kaho Takamuro, Ken-ichiro Kamei

**Affiliations:** 1 Institute for Integrated Cell-Material Sciences (WPI-iCeMS), Kyoto University, Yoshida-Ushinomiya-cho, Sakyo-ku, Kyoto, 606-8501 Japan; 2 Department of Biosystems Science, Institute for Frontier Life and Medical Sciences, Kyoto University, Shogoin-Kawara-cho, Sakyo-ku, Kyoto, 606-8397 Japan; 3 Laboratory of Cellular and Molecular Biomechanics, Graduate School of Biostudies, Kyoto University, Yoshida-Konoe-cho, Sakyo-ku, Kyoto, 606-8397 Japan; 4 Wuya College of Innovation, Shenyang Pharmaceutical University, Liaoning, 110016 People’s Republic of China; 5 Department of Pharmaceutics, Shenyang Pharmaceutical University, Liaoning, 110016 People’s Republic of China

**Keywords:** Embryonic stem cells, Differentiation

## Abstract

Hepatocyte-like cells derived from human pluripotent stem cells (hPSC-HLCs) offer an alternative to primary hepatocytes commonly used for drug screenings and toxicological tests. However, these cells do not have hepatic functions comparable to those of hepatocytes in vivo due to insufficient hepatic differentiation. Here we showed that the hepatic functions of hPSC-HLCs were facilitated by applying physiological liver temperatures during hepatic differentiation. We identified the optimal temperature by treating HLCs derived from H9 human embryonic stem cells (hESC-HLCs) at 39 °C; the 42 °C treatment caused significantly greater cell death than the 39 °C treatment. We confirmed the improvement of hepatic functions, such as albumin secretion, cytochrome P450 3A activity, and collagen production, without severe cell damage. In combination with existing hepatic differentiation protocols, the method proposed here may further improve hepatic functions for hPSCs and lead to the realization of drug discovery efforts and drug toxicological tests.

## Introduction

Hepatocytes are the major cellular component of liver tissues and play an important role in protein synthesis/storage, carbohydrate metabolism, and production of cholesterol, bile acids, and phospholipids that contribute to homeostasis in vertebrates. Evaluations of drug candidates’ safety often focus on the liver because hepatocytes metabolize chemical substances and drugs in vivo^1^. Currently, primary human hepatocytes and cell lines (e.g., HepG2 and HepaRG)^2,3^ are used, but the former is difficult to obtain from healthy donors and maintain liver functions. The latter does not represent healthy liver function due to their cancerous characteristics. Researchers have recently turned to stem cell technologies to respond to the need for alternative cost-efficient, homogenous, readily available, and viable in vitro cells for liver research.

Hepatocyte-like cells (HLCs) derived from human pluripotent stem cells (hPSC-HLCs) have considerable potential to provide optimal hepatocyte function during drug screening and toxicity tests^4^. hPSCs, such as embryonic stem cells (ESCs)^5^, and induced pluripotent stem cells (iPSCs)^6^ can differentiate into almost any type of cell and possess the ability to self-renew indefinitely. Studies have successfully induced the differentiation of hPSCs into hPSC-HLCs using growth factors and chemicals (e.g., hepatocyte growth factor [HGF], dexamethasone [DEX], and oncostatin M [OSM])^7,8^, but these hepatocytes still have fetal liver functions. In particular, cytochrome p450 3A4 (CYP3A4), which is mainly produced in the mature liver and is regarded as the most critical metabolic enzyme when optimizing drug treatments, lower in hPSC-HLCs than that in primary human hepatocytes (PHH). Alternatively, α-fetoprotein (AFP) and CYP3A7, both of which are expressed in the fetal liver, have high levels in hPSC-HLCs. These studies demonstrate the need to develop new methods to clarify the in vitro hepatic functions of hPSC-HLCs.

On exploring the hepatic physiological conditions in vivo, the liver’s temperature has been found to be higher than that of other body parts or general in vitro cell culture conditions; this is because hepatocytes work as heat-producing cells during sugar, protein, and lipid metabolism^9,10^. In contrast, as hPSC-HLCs do not have the functional levels of hepatocytes in vivo, we hypothesize that hPSC-HLCs do not produce the necessary functional temperature. Indeed, other studies showed that higher temperatures (approximately 39 °C) for in vitro cell culture promote differentiation and growth of myoblasts and neurons in other heat-producing organs, such as muscles and brain, respectively^11,12^. In contrast, it has been suggested that spermatogenesis requires low temperature^13^. Thus, each organ needs to be exposed with an appropriate temperature to maximize its function; there is a clear need to identify the proper temperature to functionalize hPSC-HLCs.

Here we demonstrated the heat-induced functionalization of hPSC-HLCs and elucidated the functionalization process’s underlying mechanism by heat stimulation. We concluded that mild heat treatment at 39 °C during the hepatocyte-maturation process, but not higher temperatures such as 42 °C, was able to functionalize hPSC-HLCs, such as albumin secretion, exogenous substances’ uptake, and excretion and metabolic activities, such as of the CYP family (e.g., CYP3A4 and CYP3A7), after 12-day treatment. To further understand the molecular mechanisms, RNA sequencing (RNA-seq) and molecular biological analyses were conducted and revealed that heat treatment induced the critical genes associated with not only molecular chaperons of heat shock proteins (HSPs) but also extracellular matrices (ECMs) associated with liver structures and functions.

## Results

### hESC-HLCs survive during treatment at 39 °C

**Figure 1:**
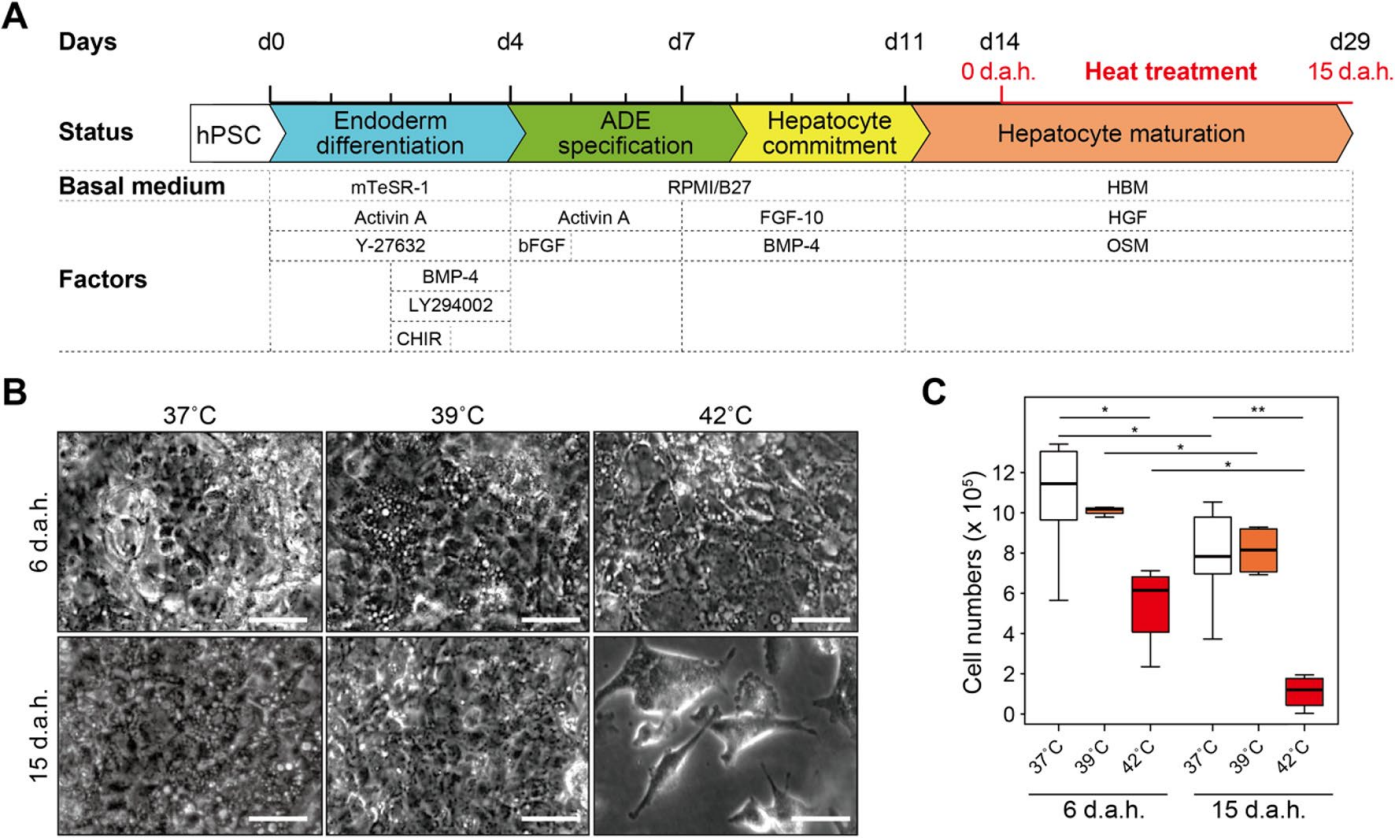
Functionalization of hepatocyte-like cells derived from hESCs by heat treatment. (**A**) Schematic representation of the protocol to functionalize hESC-HLCs. d.a.h. represents days after heat treatment. Initially, hPSCs were treated with mTeSR-1 medium supplemented with combinations of Activin A, BMP-4, CHIR99021 (CHIR), LY294002, and Y-27632 to induce definitive endoderm (DE) differentiation until Day 4 (d4). Then, cells were treated with Activin A in RPMI medium supplemented with B27 for anterior definitive endoderm (ADE) specification until Day 8. As the next step, cells were treated with BMP-4 and FGF-10 for inducing hepatocyte commitment in RPMI medium supplemented with B27 until Day 11. Finally, cells were treated with oncostatin M (OSM) and hepatocyte growth factor (HGF) in basal hepatocyte medium for hepatocyte maturation. On day 14, cells were incubated at 37, 39, or 42 °C, and half the total amount of the medium was changed every day. (**B**) Microscopic images of hESC-HLCs treated with 37, 39, and 42 °C at 6 and 15 d.a.h. Scale bars represent 50 µm. (**C**) Box plots showing the cell numbers of living hESC-HLCs after heat treatments at 37, 39, and 42 °C at 6 and 15 d.a.h. (n = 4). Centerlines of box plots indicate medians; box limits indicate the 25th and 75th percentiles as determined by *R* software; whiskers extend 1.5 times the interquartile range from the 25th and 75th percentiles; *p < 0.05, **p < 0.01.

To prove that higher temperature than normal body temperature (37 °C) functionalize hESC-HLCs, we treated the hepatic progenitor cells derived from WA09 (H9) hESCs with higher temperatures at 37, 39, and 42 °C during the hepatic differentiation process^14^ from day 14 (Fig. 1A). 42 °C-treated cells at 15 days after heat treatment (d.a.h.) showed apparent cell detachment from a cell-culture dish, while for 39 °C-treated cells, there was no notable detachment and morphological change compared with that in 37 °C-treated cells (Fig. 1B). During hepatic differentiation, cells in all tested conditions showed reduced cell numbers (n = 4, Fig. 1C). Therefore, we used only the 37 °C and 39 °C-treatment to facilitate the hepatic functions of hESC-HLCs.

### 39 °C-treatment during differentiation phase activates CYP3A activity of hESC-HLCs

**Figure 2:**
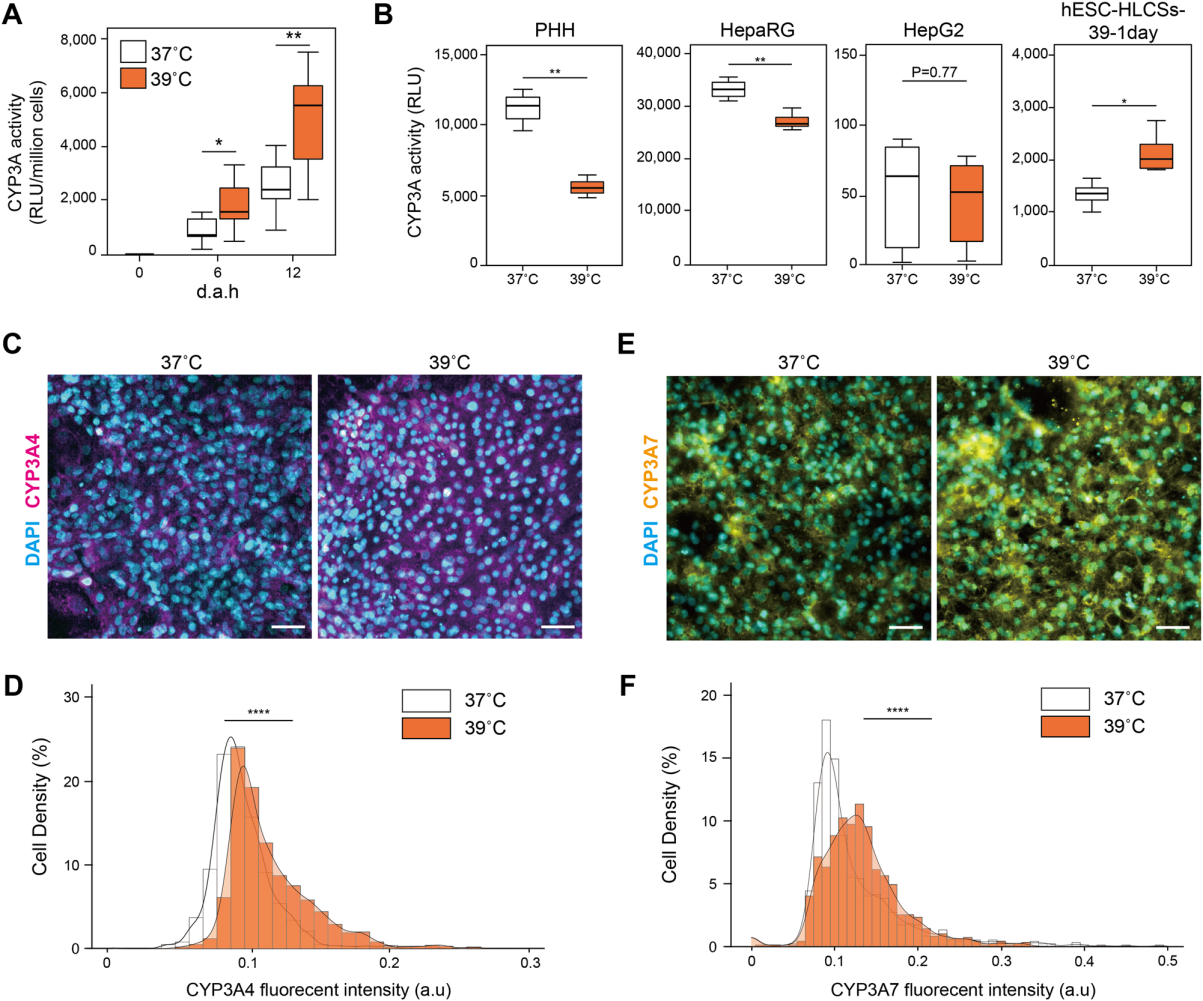
Evaluation of expression of hepatic metabolic CYP enzymes in hESC-HLCs with heat treatments. (**A**) Bioluminescent-based CYP3A activity assay to confirm CYP3A activities during the hepatic differentiation process (n = 7). (**B**) Bioluminescent-based CYP3A activity assay to confirm CYP3A activities of hepatocytes cultured under 39 °C for a day (n = 3). (**C,D**) Immunofluorescent micrographs (**C**) and single-cell profiling (**D**) of CYP3A4 in 37 °C- and 39 °C-treated hESC-HLCs at 12 d.a.h. (n = 3). (**E,F**) Immunofluorescent micrographs (**E**) and single-cell profiling (**F**) of CYP3A7 in 37 °C- and 39 °C-treated hESC-HLCs at 12 d.a.h. (n = 3). DAPI was used for nuclei staining. Scale bars = 50 µm. *p < 0.05, **p < 0.01, ****p < 0.001, each plot and error bar represents a mean ± SD.

To investigate the contribution of heat treatment to the hepatic metabolic activities, we conducted the bioluminescent-based CYP3A activity assay to confirm CYP3A activities during the hepatic differentiation process (Fig. 2A). Both 37 °C- and 39 °C-treated cells showed increased CYP3A activities, and the CYP3A activity of the 39 °C-treated cells significantly increased at 6 d.a.h. (p = 0.045, n = 7) and 12 d.a.h. (p < 0.001, n = 7). To examine whether such heat treatment facilitates the hepatic metabolic activities, we also treated generally used hepatocytes (i.e., PHH, differentiated HepaRG, and HepG2 cells). Because PHH and differentiated HepaRG cells would lose their functions in in vitro cell culture, these cells were treated for 24 h. Interestingly, the CYP3A activities of PHH and HepaRG were significantly decreased after heat treatment at 39 °C for 24 h (p < 0.01, n = 3, Fig. 2B, and S1A). In contrast, HepG2 cells did not show a significant change in CYP3A activities (p = 0.77, n = 3), but the 12 d.a.h. treatment significantly decreased CYP3A protein expression (p < 0.05, n = 3, Fig. S1B). We also tested to a-day treatment at 39 °C for hESC-HLCs, which had already been cultured at 37 °C until 12 d.a.h., (hESC-HLCs-39-1 day). Notably, hESC-HLCs-39-1 day significantly increased CYP3A activity (p < 0.05, n = 3, Fig. 2B). These results indicate that this increase in CYP3A activity by the 39 °C treatment is specific during the hepatic differentiation process but not for fully differentiated hepatocytes, such as PHH.

The used CYP3A activity assay can detect both CYP3A4 and CYP3A7 and cannot distinguish them. To elucidate which CYP3A4 or CYP3A7 showed the activities in 39 °C-treated cells, fluorescent immunocytochemistry followed by quantitative single-cell profiling was performed (n = 3, Fig. 2C–F). As a result, 39 °C-treated cells expressed both CYP3A4 and CYP3A7 proteins more than the 37 °C-treated cells (Fig. 2D,F, S1E, and S1G). Thus, it is likely that both CYP3A4 and CYP3A7 showed metabolic activities. We also performed quantitative RT-PCR analysis to compare the mRNA expressions of *CYP3A4* and *CYP3A7* as well as glyceraldehyde-3-phosphate dehydrogenase (*GAPDH*) as a housekeeping gene. Because the cycle threshold (Ct) values of *GAPDH* in the 37 °C- and 39 °C-treated cells did not show a significant difference (n = 3, Fig. S1C), the Ct values of *GAPDH* could be used as a loading control in quantitative RT-PCR. Consequently, we could not see a difference in *CYP3A4* and *CYP3A7* gene expressions between both temperatures (Fig. S1D and S1F). The discrepancy among protein expression, metabolic activity and mRNA expression could reflect the post-translational modification caused by heat treatment, but further investigation is necessary.

Furthermore, to investigate the effects on the other CYP family, the expression of hepatic CYP genes (e.g., *CYP1A2*, *CYP2A6*, *CYP2C8*, *CYP2D6*, *CYP2E1*, and *CYP7A1*) were examined using quantitative RT-PCR (n = 3, Fig. S1H). The expressions of *CYP2A6*, *CYP2D6*, *CYP2E1*, and *CYP7A1* continuously increased during hepatic differentiation process in the 37 °C- and 39 °C-treated hESC-HLCs. Moreover, the expressions of *CYP1A2, CYP2A6*, *CYP2C8*, and *CYP7A1* were elevated in only the 39 °C-treated cells.

### 39 °C-treatment facilitates’ hepatic functions of hESC-HLCs

**Figure 3:**
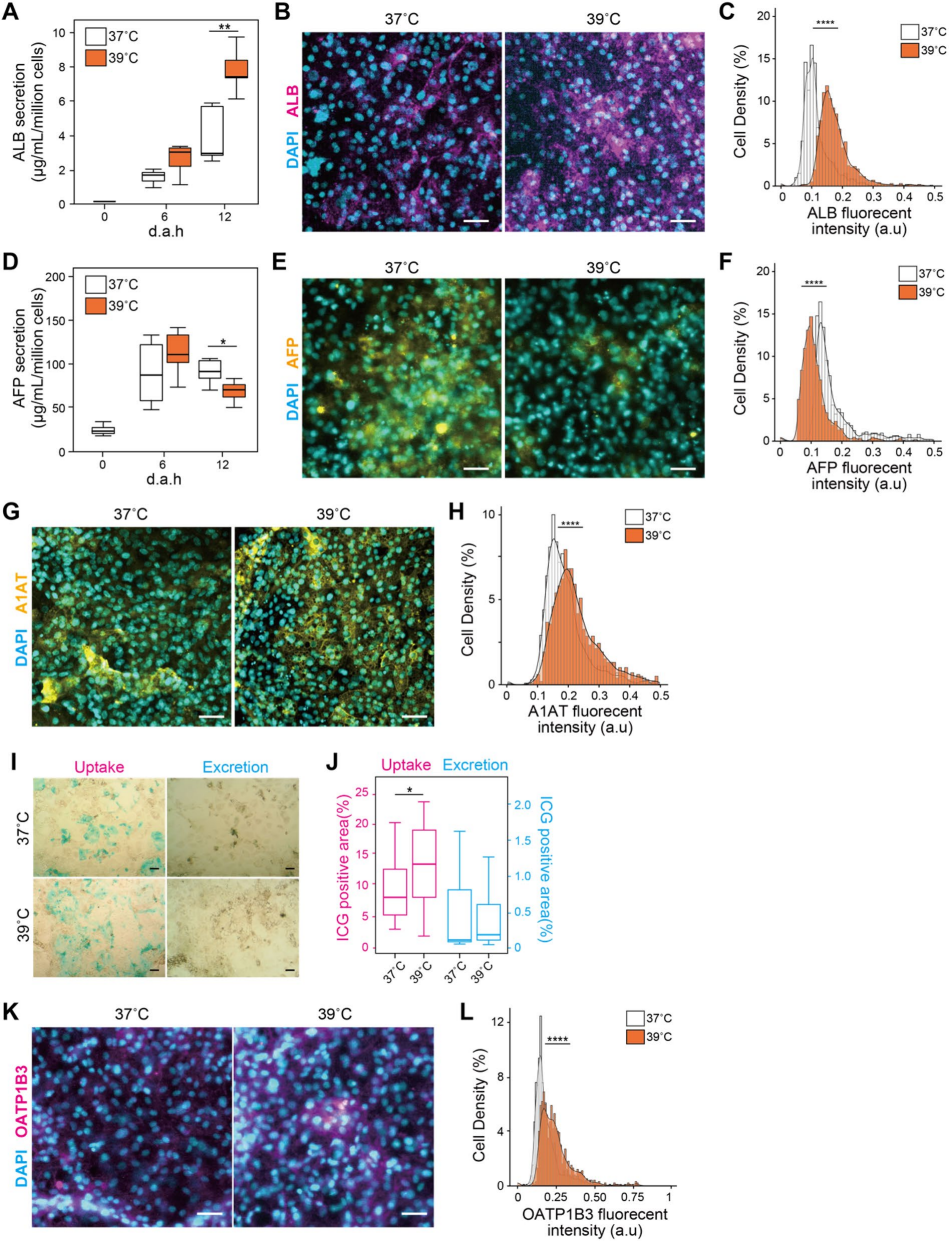
Heat treatment at 39 °C facilitates hepatic functions of hESC-HLCs. (**A**) Albumin (ALB) secretion from 37- to 39 °C-treated hESC-HLCs at 12 d.a.h. measured by ELISA. (**B,C**) Immunofluorescent micrographs (**B**) and single-cell profiling (**C**) of ALB in 37 °C- and 39 °C-treated hESC-HLCs at 12 d.a.h. (n = 3). (**D**) α-fetoprotein (AFP) secretion from 37- to 39 °C-treated hESC-HLCs at 12 d.a.h. measured by ELISA. (**E,F**) Immunofluorescent micrographs (**E**) and single-cell profiling (**F**) of AFP in 37 °C- and 39 °C-treated hESC-HLCs at 12 d.a.h. (n = 3). (**G,H**) Immunofluorescent micrographs (**G**) and single-cell profiling (**H**) of α1-anti trypsin (A1AT) in 37 °C- and 39 °C-treated hESC-HLCs at 12 d.a.h. (n = 3). (**I,J**) Micrographs (**I**) and the percentiles of positive area (**J**) of 37 °C- and 39 °C-treated hESC-HLCs at 12 d.a.h., stained with indocyanine green (ICG) (n = 5) Scale bars = 50 µm. (**K,L**) Immunofluorescent micrographs (**K**) and single-cell profiling (**L**) of organic anion transporting polypeptide 1B3 (OATP1B3) in 37 °C- and 39 °C-treated hESC-HLCs at 12 d.a.h. (n = 3). DAPI was used for nuclei staining. Scale bars = 50 µm. In all panels, where applicable, center lines of box plots indicate medians; box limits indicate the 25th and 75th percentiles as determined by *R* software; whiskers extend 1.5 times the interquartile range from the 25th and 75th percentiles; *p < 0.05, **p < 0.01, ***p < 0.005, ****p < 0.0001; each plot and error bar represents a mean ± SD.

Besides the hepatic metabolic activities, we also evaluated the hepatic functionalities, such as albumin (ALB) expression/secretion (Fig. 3A–C, S2A, and S2B), α-fetoprotein (AFP) expression/secretion, (Fig. 3D–F, S2C, and S2D), α1 anti-trypsin (A1AT, or called SERPINA1) (Fig. 3G,H, S2E, and S2F), uptake/excretion of exogenous substances (Fig. 3I–J, and S2G–K), and glycogen storage (Fig. S2L, and S2M). Among them, we were able to observe the difference of ALB expression/secretion and uptake/excretion of exogenous substances at the 39 °C treatment.

The 37 °C- and 39 °C-treated hESC-HLCs secreted albumin (ALB) at 6 and 12 d.a.h., and 39 °C-treated cells secretion was significantly higher than that of 37 °C-treated cells at 12 d.a.h. (p = 0.002, n = 5, Fig. 3A). Compared with 37 °C-treated hESC-HLCs, 39 °C-treated cells had increased ALB expression (p < 0.001, n = 3, Fig. 3B,C, and S2B) and mRNA levels (p = 0.007, n = 3, Fig. S2A). Similarly, the protein and mRNA expressions of α1 anti-trypsin (A1AT, or called SERPINA1), one of liver maturation markers, were increased in 39 °C-treated cells (p < 0.001, n = 3, Fig. 3G,H, S2E, and S2F). In contrast, in the case of AFP, a biomarker of embryonic hepatic development, both the secretion and protein level were decreased in 39 °C-treated cells (p = 0.032, n = 6, Fig. 3D,E, and S2D), but the mRNA levels were not significantly different (p = 0.10, n = 3, Fig. S2C). These results suggest that 39 °C treatment facilitated the maturation process of hepatic differentiation.

To confirm their abilities to uptake and excrete exogenous substances, indocyanine green (ICG)^15^ was used to visualize the capable cells (n = 5, Fig. 3I,J). The ICG uptake levels of 39 °C-treated cells were significantly higher than those of 37 °C-treated cells at 12 d.a.h. (p = 0.039), but the excretion levels were not at 13 d.a.h. (p = 0.616). We found that one of the primary transporters of ICG, OATP1B3 was increased in 39 °C-treated cells (n = 3, Fig. 3K,L, S2G, and S2H), but not the other primary transporting NTCP (n=3, Fig. S2I–K).

### Transcriptional analysis identified gene expressions of 39 °C-treated hESC-HLCs

**Figure 4:**
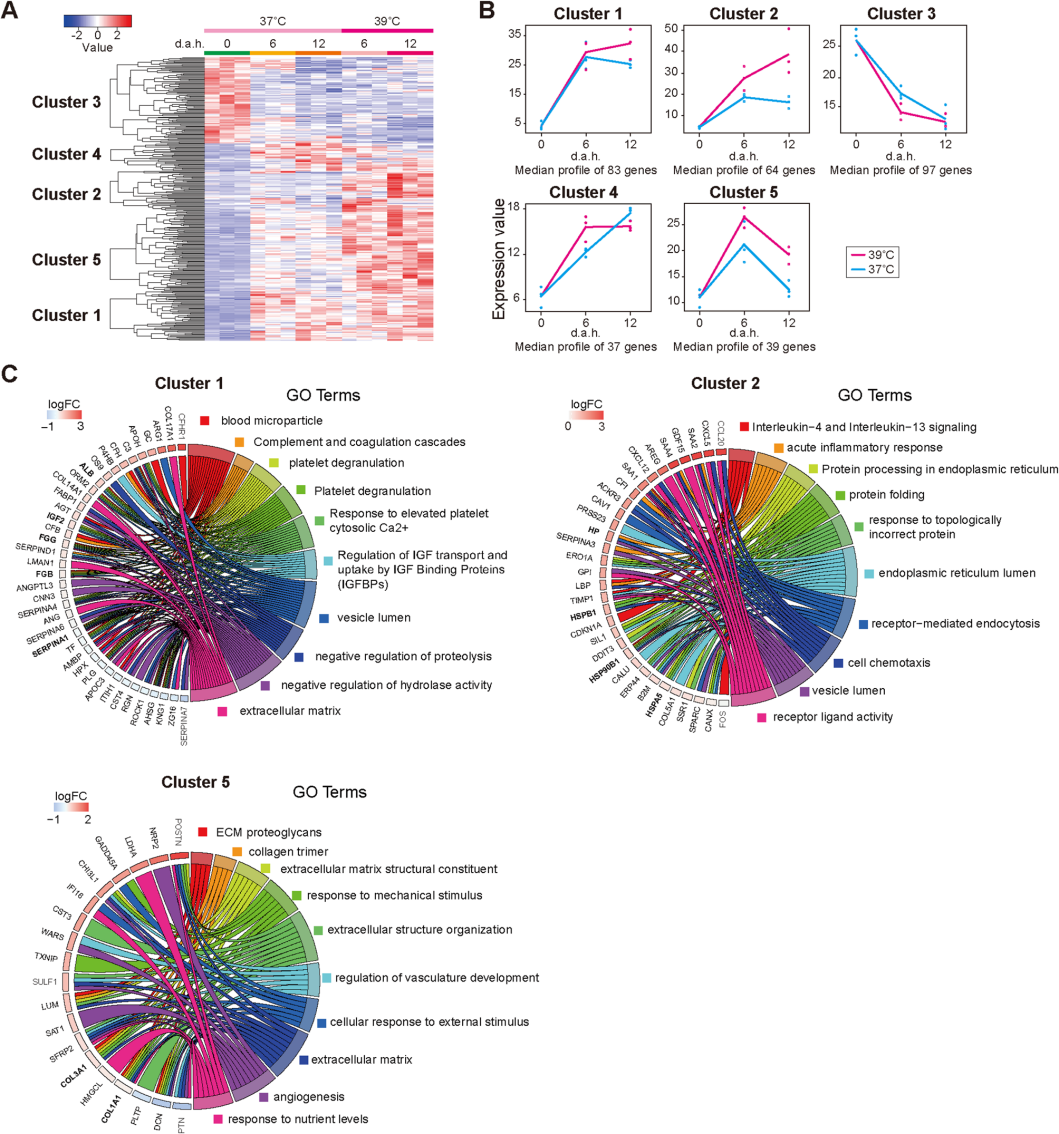
Global transcriptional analysis to identify the specific gene signatures in hESC-HLCs with heat treatments at 37 °C and 39 °C. (**A**) Hierarchical clustering and heat map of the differentially expressed genes in hESC-HLCs with heat treatments at 37 °C and 39 °C. (**B**) Typical plots of five clusters identified in (**A**). Each dot represents the median of expression values of each sample. (**C**) Chord diagram presenting enriched GO clusters for the differentially expressed genes hESC-HLCs with heat treatment at 37 °C and 39 °C. In each chord diagram, enriched GO clusters are shown (right), and genes contributing to this enrichment shown (left). Each cluster was found in (**A**).

To identify the genes affected by heat treatment at 39 °C, we performed a time-course RNA-seq analysis at three time-points with three biological replicates (0, 6, and 12 d.a.h.) using maSigPro^16^, and identified 320 significant differentially expressed genes (DEGs, Table S1). Hierarchical clustering based on the identified genes was categorized into 5 clusters (Fig. 4A,B, and S3A). Clusters 1 and 4 have DEGs which the expression levels of 39 °C-treated hESC-HLCs were similar to those of 37 °C-treated cells until 6 d.a.h., and were significantly higher from 6 to 12 d.a.h. DEGs in Cluster 2 showed that both 37 °C- and 39 °C-treated hESC-HLCs increased the DEGs, but 39 °C-treated hESC-HLCs have significantly higher expression during the hepatic differentiation process. In contrast, Cluster 3 has DEGs, which continuously decreased their expression during hepatic differentiation, but does not show the difference between the temperatures. DEGs in Cluster 5 showed increased from 0 to 6 d.a.h., once, but decreased from 6 to 12 d.a.h.. Furthermore, 39 °C-treated hESC-HLCs showed significantly higher expression of the DEGs in Cluster 5, compared with 37 °C-treated cells for the tested period.

To clarify what type of GO term or pathway the gene altered in each cluster, Gene Ontology (GO) enrichment, KEGG, and Reactome^17^ analyses were performed (Fig. 4C, S3–S5, and Table S2). Cluster 1 had 83 genes, and particularly, complement factor H related 1 (*CFHR1*), *ALB*, insulin-like growth factor 2 (*IGF2*), fibrinogen beta chain (*FGB*), and fibrinogen gamma chain (*FGG*) showed high expression. These genes showed biological terms associated with liver functions such as “blood microparticle”^18,19^ and “regulation of insulin-like growth factor transport and uptake by insulin-like growth factor binding proteins”, with high enrichment ratio (FDR < 0.05). These results suggested that the genes in Cluster 1 have roles of liver functions activated by heat treatment.

Cluster 2 had 64 genes, including haptoglobin (*HP*), heat shock protein 90 kDa beta member 1 (*HSP90B1*), heat shock protein family A (HSP70) member 5 (*HSPA5*), and heat shock protein beta-1 (*HSPB1*). These genes were associated with the terms “Protein processing in endoplasmic reticulum” with high enrichment ratios (FDR < 0.05).

Cluster 5 had 39 genes with higher expression in 39 °C-treated hESC-HLCs than in 37 °C-treated hESC-HLCs, such as collagen type I alpha 1 chain (*COL1A1*). These genes are involved in ECM construction, such as “extracellular matrix structural constituent” (FDR < 0.05).

### 39 °C-treatment activates collagen production of hESC-HLCs

**Figure 5:**
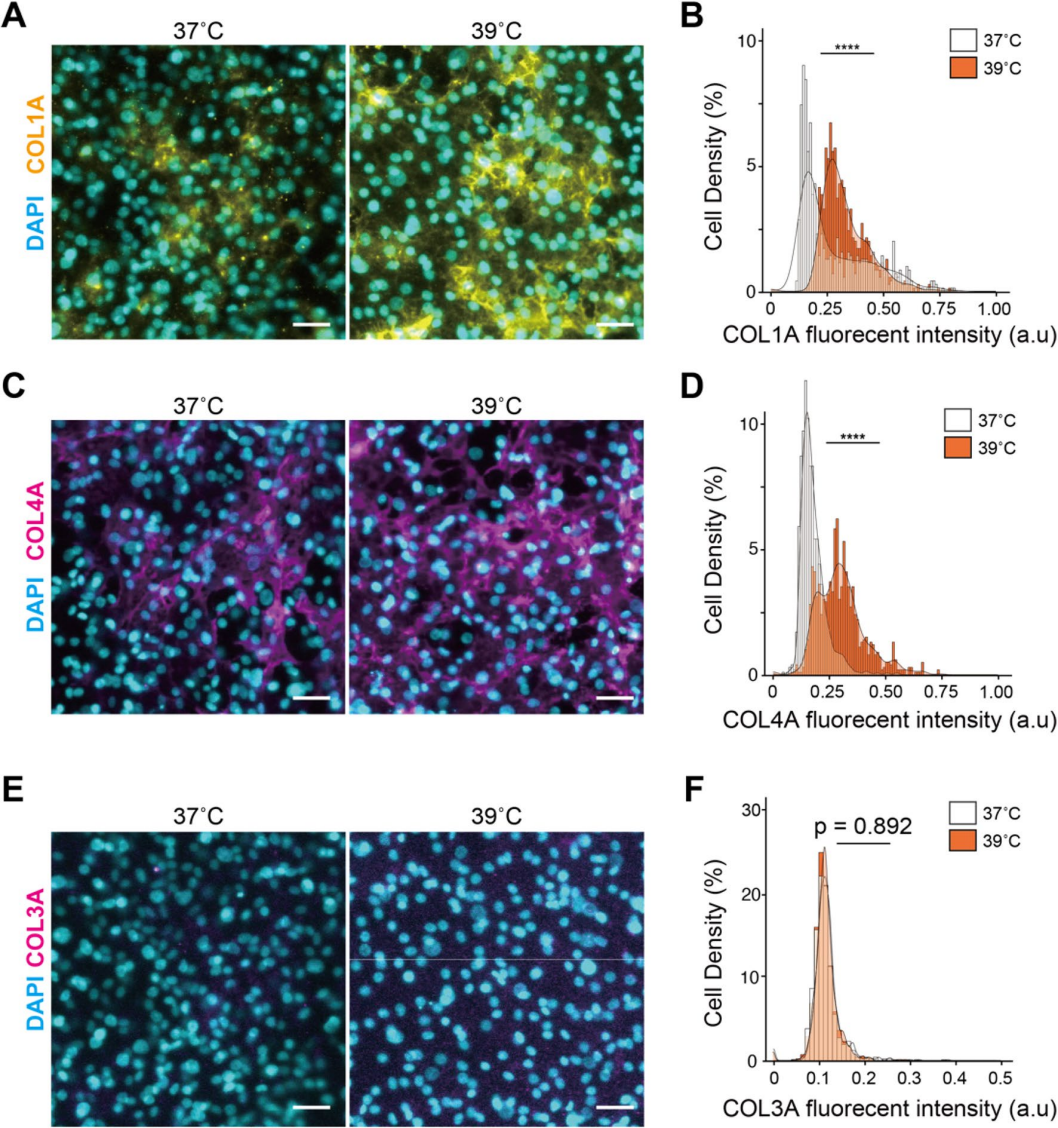
Evaluation of expression of collagen type I (COL1A), collagen type IV (COL4A), and collagen type III (COL3A) in hESC-HLCs with heat treatments. (**A,B**) Immunofluorescent micrographs (**A**) and single-cell profiling (**B**) of COL1A in 37 °C- and 39 °C-treated hESC-HLCs at 12 d.a.h. (n = 3). (**C,D**) Immunofluorescent micrographs (**C**) and single-cell profiling (**D**) of COL4A in 37 °C- and 39 °C-treated hESC-HLCs at 12 d.a.h. (n = 3). (**E,F**) Immunofluorescent micrographs (**E**) and single-cell profiling (**F**) of COL3A in 37 °C- and 39 °C-treated hESC-HLCs at 12 d.a.h. (n = 3). In all panels, where applicable, center lines of box plots indicate medians; box limits indicate the 25th and 75th percentiles as determined by *R* software; whiskers extend 1.5 times the interquartile range from the 25th and 75th percentiles; ****p < 0.0001; each plot and error bar represents a mean ± SD. DAPI was used for nuclei staining. Scale bars = 50 µm.

Collagens are known to be a constitutive ECM of the liver and play various roles not only in liver development but also in diseases^20^. Collagen type I (COL1A) and collagen type IV (COL4A) have been reported to promote hepatocytes’ functions, such as CYP3A4, CYP3A7, and albumin^21^. Our mRNA-seq results showed that heat treatment at 39 °C increased the expression of *COL1A1* and *COL3A1*, which encode COL1A and COL3A, respectively. Therefore, we performed fluorescence immunocytochemistry and quantitative single-cell profiling of COL1A and COL3A, and COL4A which was not detected as DEGs by RNA-seq but is involved in the liver constitution. The expression of COL1A and COL4A was observed in both 37 °C- and 39 °C-treated hESC-HLCs but was significantly higher in 39 °C-treated cells than in 37 °C-treated cells (p < 0.001, n = 3, Fig. 5A–D, S6A, and S6B). In contrast, COL3A had a low expression level in both 37 °C- and 39 °C-treated cells, and there was no significant difference (p = 0.89, n = 3, Fig. 5E,F, and  S6C). Thus, we confirmed that 39 °C-treated hESC-HLCs elevated the expression of COL1A and COL4A.

## Discussion

Fully functional human hepatocytes have a great potential for applications in drug screening and toxicological tests as well as regenerative medicine. Here, we present a new method to functionalize HLCs derived from hPSCs by providing heat treatment at 39 °C during hepatic differentiation in combination with the conventional hepatic differentiation protocol with the use of chemicals and growth factors^14^, but not a higher temperature such as 42 °C, and with chemical treatments. In contrast, we found that this heat treatment was able to functionalize hPSC-HLCs, but not fully differentiated hepatocytes, such as PHH and differentiated HepaRG. Therefore, such heat treatment is required during hepatic differentiation.

Hepatic metabolic activity by the CYP family is the one of most important features of hepatocytes. We found that the CYP3A activity of hPSC-HLCs was increased in 39 °C-treated cells. Single-cell profiling based on immunofluorescent microscopic images also showed that 39 °C-treated cells had increased CYP3A7/CYP3A4 expressions.

In addition to hepatocyte-associated genes, the genes of several molecular chaperones HSPs (Heat shock protein [HSP] 90 beta family member 1 [*HSP90B1*], HSP family A [HSP70] member 5 [*HSPA5*], and HSP family B [small] member 1 [*HSPB1*]) were induced by heat treatment at 39 °C. Molecular chaperons generally work for protein folding, liver development^22^, and disease progression^23^. In particular, HSP90B1 and HSPA5 (or called glucose-regulated protein 78 [GRP78]) were expressed in the liver, according to the Human Protein Atlas^24^. HSPB1 expression has been reported in a variety of organs, including the liver. In terms of cellular status, which we observed, 39 °C-treated hESC-HLCs showed similar cell numbers as 37 °C-treated hESC-HLCs. In contrast, the CYP3A4 activity was upregulated, although its gene expression did not change. These results suggest that molecular chaperons support CYP3A4 folding, resulting in the promotion of its activity. Thus, the upregulated chaperons supported protein folding rather than cancer- or disease-like status.

Although we improved the functionality of hepatocytes derived from hPSCs, there is still a need for improvement. The obtained hepatocytes have not reached the adult liver stage because CYP3A7 and AFP, which are expressed during the fetal hepatic developmental process^25,26^, were expressed in 39 °C-treated hESC-HLCs. Therefore, additional treatments need to be established for the further maturation of hepatocytes. While chemical environmental cues have been studied for functionalized hPSC-HLCs, physical cues are still largely uncovered. Recently, to recapitulate liver-like cell aggregates, liver organoid technology using hPSCs showed strong potential for application in drug discovery and regenerative medicine^27^. However, even such organoids, which are generally obtained with chemical environmental cues, do not reach the liver’s functional levels in vivo. Thus, physical environmental cues, such as shown in this study, would facilitate the hepatic functions of hepatocytes and liver organoids. In particular, the thermal conditions required for liver functionalization are quite interesting but not fully understood. We could not determine whether hepatocytes generate the heat first or whether high environmental temperatures allow hepatocytes for heat generation. We applied external heat treatment on hPSC-HLCs and confirmed that hPSC-HLCs were functional, but we could not confirm whether hPSC-HLCs generated heat or not, because if so, the temperature changes would be very small. Recently, intracellular thermal biosensors based on genetically engineered green fluorescent proteins have been established^28^, and the use of such biosensors would be beneficial to investigate the mechanisms of thermal regulation for liver functionalization.

In conclusion, we showed the physiologically relevant heat condition at 39 °C allows functionalizing hESC-HLCs, such as albumin secretion, CYP3A activities, and collagen productions, without severe cell damages. Since this approach is straightforward and does not require any special instruments, it would be rapidly used for practical applications of functionalized hPSC-HLCs for drug discovery and toxicological tests.

## Materials and methods

### hESC culture

hESCs were used according to the guidelines provided by the ethical committee of Kyoto University (Approved# ES3-9). WA09 (H9) (RRID:CVCL_9773, hPSCreg Name WAe009-A) hESCs used in this study were purchased from WiCell Research Institute (Madison, WI, USA). Before culturing, hESC-certified Matrigel (Corning, Corning, NY, USA) was diluted with Dulbecco’s modified Eagle medium (DMEM)/F12 medium (Sigma-Aldrich, St. Louis, MO, USA) at a 1:75 (v/v) ratio and coated onto a culture dish. The Matrigel was incubated in the culture dish for 24 h at 4 °C. Then, excess Matrigel was removed, and the coated dish was washed with fresh DMEM/F12 medium. We used mTeSR-1-defined medium (Stem Cell Technologies, Vancouver, Canada) for daily culturing of hPSCs. For passaging, cells were dissociated with TryPLE Express (Thermo Fisher Scientific, Tokyo, Japan) for 3 min at 37 °C and then harvested. A cell strainer was used to remove undesired cell aggregates from the cell suspension, and cells were then centrifuged at 200×*g* for 3 min and resuspended in mTeSR-1 medium. Live/dead cells were counted using a NucleoCounter NC-200 (Chemetec, Baton Rouge, LA, USA). We used mTeSR-1 medium containing 10 µM of the ROCK inhibitor Y-27632 (Wako, Osaka, Japan) to prevent apoptosis of dissociated hPSCs on day 1. On subsequent days, we used mTeSR-1 medium without the ROCK inhibitor with daily medium changes.

### Hepatic differentiation from hPSC

Before inducing differentiation, we coated a cell-culture dish with 0.1% gelatin in phosphate-buffered saline (PBS, Thermo Fisher Scientific) for 30 min at 25 °C. We then aspirated the gelatin solution and introduced a DMEM/F12 medium (Sigma-Aldrich) onto the culture dish for serum coating at 37 °C for 24 h. The medium was supplemented with 10% (v/v) fetal bovine serum (Cell Culture Bioscience, Tokyo, Japan), penicillin/streptomycin (Wako), and 100 µM β-mercaptoethanol (Sigma-Aldrich). The coated dish was then rinsed with fresh medium.

Cultured hPSCs were washed with PBS and treated with TryPLE Express at 37 °C for 5 min, followed by the addition of basal medium and the transfer of the cell suspension into a 15 mL tube to induce endoderm differentiation. Cells were centrifuged at 200×*g* for 3 min, the supernatant was removed, and then the cells were resuspended in mTeSR-1 medium supplemented with 10 µM Y-27632 and 100 ng mL^−1^ activin A (Wako), plated on a serum-coated culture dish, and cultured in a humidified incubator at 37 °C with 5% CO_2_ for 24 h. At the end of day 1, the medium was replaced with fresh mTeSR-1 medium supplemented with 10 µM Y-27632 and 100 ng mL^−1^ activin A and cultured for another 24 h. On day 2, the medium was replaced with mTeSR-1 medium supplemented with 10 µM Y-27632, 100 ng mL^−1^ activin A, 10 ng mL^−1^ bone morphogenetic protein-4 (BMP-4) (R&D Systems), 10 µM LY294002 (Cayman Chemical, Ann Arbor, MI, USA), and 3 µM CHIR99021 (Stemgent, Cambridge, MA, USA), and cells were incubated for 24 h. On day 3, the medium was replaced with mTeSR-1 medium supplemented with 10 µM Y-27632, 100 ng mL^−1^ activin A, 10 ng mL^−1^ BMP-4, and 10 µM LY294002, and cells were incubated for 24 h. On day 4, the medium was replaced with Roswell Park Memorial Institute (RPMI) medium (Thermo Fisher Scientific) supplemented with B-27 (Thermo Fisher Scientific), 100 ng mL^−1^ activin A, and 100 ng mL^−1^ basic fibroblast growth factor (bFGF), and cells were incubated for 24 h. To induce ADE specification, cells were treated with RPMI medium supplemented with 50 ng mL^−1^ activin A, with daily medium changes for three days. Cells were then treated with RPMI medium supplemented with 20 ng mL^−1^ BMP-4 and 10 ng mL^−1^ FGF-10 (R&D systems), with daily medium changes for four days. On day 12, the medium was replaced with hepatocyte-maturation medium (hepatocyte basal medium (Lonza, Basel, Switzerland) supplemented with 30 ng mL^−1^ oncostatin M (R&D Systems), 50 ng mL^−1^ HGF (PeproTech, Rocky Hill, NJ), and 25 mM HEPES (Wako) to induce maturation of the differentiated hepatocytes. On day 14, the cells were incubated at 37 °C, 39 °C, or 42 °C, and half the total amount of medium was changed every day. To avoid evaporation of the medium, which might lead to artifacts in hepatic differentiation and functional measurements, the amounts of the medium were monitored and maintained at the original volume (1 mL in each well of a 24-well plate) for the differentiation process.

### CYP3A activity assay

We used a cytochrome P450 3A (CYP3A) Assay and Screening System with Luciferin-IPA (Promega, Madison, MI, USA) to assess CYP3A activity. Samples were treated with a luciferin-IPA substrate (1:1000) in hepatocyte-maturation medium at 0, 6, and 12 d.a.h. of either 37 °C or 39 °C. We collected the medium after 1 h and added Luciferin Detection Reagent (Promega). After 15 min, the CYP3A activity was measured in each sample with a Synergy HTX multi-mode reader (BioTek Instruments, Inc. Winooski, VT, USA). The activity was normalized based on the total number of cells.

### ELISA for human albumin and α-fetoprotein

The medium cultured with cells were collected and stored at − 80 °C until use. The concentrations of albumin and α-fetoprotein secreted into the medium were measured using human albumin ELISA kit (Abcam, Cambridge, Cambridgeshire, UK, ab179887) and human α-fetoprotein ELISA kit (Proteintech, Tokyo, JAPAN), following the manufacture’s protocol.

### Single-cell profiling based on microscopic images

Following the microscopic image acquisition, the CellProfiler software (Broad Institute of Harvard and MIT, Version 4.1.2) was used to identify cells with Otsu’s method. The fluorescence signals of individual cells were quantified automatically. Single-cell profiling was calculated based on 1000 cells randomly selected from fluorescent microscopic images.

### RNA amplification and sequencing

Precisely 40 ng of total RNA was diluted with 9 μL of RNase free water, then mixed with VN primer (Oxford NANOPORE Technologies, UK) and 1 μL of 10 mM dNTPs (New England Biolabs Inc. Ipswich, Massachusetts, USA), and incubated at 65 °C for 5 min to prepare the cDNA library. Separately, 4 μL of 5× RT Buffer (Thermo Fisher Scientific), 1 μL of RNaseOUT (Thermo Fisher Scientific), 1 μL of Nuclease-free water, and 2 μL of Strand-Switching Primer (Oxford NANOPORE Technologies) was mixed as the strand-switching buffer. The two solutions were mixed at 42 °C for 2 min; then, 1 μL of Maxima H Minus Reverse Transcriptase (Thermo Fisher Scientific) was added. The mixture was incubated at 42 °C for 90 min, 85 °C for 5 min, and stored at 4 °C until use as the cDNA library. Exactly 5 μL aliquot of the cDNA library solution was mixed with 25 μL of 2× LongAmp Taq Master Mix (New England Biolabs Inc.), 1.5 μL of Barcode Primers (Oxford NANOPORE Technologies), and 18.5 μL of nuclease-free water. PCR was performed (95 °C for 30 s, 18 cycles of 95 °C for 15 s, 62 °C for 15 s and 65 °C for 50 s, and then 65 °C for 6 min) to barcode the cDNA for multiplexing. PCR products were stored at 4 °C until use. Then, 1 μL of NEB Exonuclease 1 (New England Biolabs Inc.) was added before incubation at 37 °C for 15 min, followed by incubation at 80 °C for 15 min. Using Agencourt AMPure XP beads (BECKMAN COULTER Life Sciences, Indianapolis, IN), amplified DNA was purified and collected in 12 μL of Elution Buffer (Oxford NANOPORE Technologies). BioAnalyzer 2100 with High Sensitivity DNA Kit (Agilent Technologies) was used to evaluate barcoded cDNA’s amount and quality. Then, 50 fmol of the barcoded cDNA was incubated with 1 μL of Rapid Adapter to make up a total volume of 11 μL, which was incubated for 5 min at 25 °C. For Nanopore sequencing, 12 μL of the prepared DNA library was mixed with 37.5 μL of Sequencing Buffer (Oxford NANOPORE Technologies) and 25.5 μL of Loading Buffer (Oxford NANOPORE Technologies). This solution was applied to the Nanopore Flow Cell (v9.4.1) and run for 24 h.

Additional materials and methods are available in the Supplementary Information.

## Supplementary Information

Supplementary Information.

## Data Availability

The mRNA-seq data have been deposited in the NCBI Gene Expression Omnibus under Accession Number GSE172227.
