# Supplementary material for: In vitro culture at 39 °C during hepatic maturation of human ES cells facilitates hepatocyte-like cell functions
Source: Sci Rep. 2022 Mar 25;12:5155. doi: 10.1038/s41598-022-09119-7 (PMC8956733; doi:10.1038/s41598-022-09119-7)
Supplement: Supplementary file 1 — Supplementary Information. [file 41598_2022_9119_MOESM1_ESM.docx]

**Supporting Information**

***In vitro* culture at** **39°C during hepatic maturation of human ES cells facilitates hepatocyte-like cell functions**

Satoshi Imamura^1^, Koki Yoshimoto^1,2,3^, Shiho Terada^1^, Kaho Takamuro^1^, and Ken-ichiro Kamei^1,4,5,^*

^1^Institute for Integrated Cell-Material Sciences (WPI-iCeMS), Kyoto University, Yoshida-Ushinomiya-cho, Sakyo-ku, Kyoto 606-8501, Japan

^2^Department of Biosystems Science, Institute for Frontier Life and Medical Sciences, Kyoto University, Shogoin-Kawara-cho, Sakyo-ku, Kyoto 606-8397, Japan

^3^Laboratory of Cellular and Molecular Biomechanics, Graduate School of Biostudies, Kyoto University, Yoshida-Konoe-cho, Sakyo-ku, Kyoto 606-8397, Japan

^4^Wuya College of Innovation, Shenyang Pharmaceutical University, Liaoning 110016, People’s Republic of China

^5^Department of Pharmaceutics, Shenyang Pharmaceutical University, Liaoning 110016, People’s Republic of China

*To whom correspondence may be addressed.

**Email***:* [kamei.kenichiro.7r@kyoto-u.ac.jp](mailto:kamei.kenichiro.7r@kyoto-u.ac.jp).

Address: Yoshida-Ushinomiya-cho, Sakyo-ku, Kyoto, 606-8501, JAPAN

Tel: +81-75-753-9774/Fax: +81-75-753-9761

**Materials and methods**

**Hepatocytes culture.** Primary human hepatocytes (PHH) were purchased from SEKISUI MEDICAL CO., LTD. PHH were seeded on 0.1% collagen type I-coated plate with OptiPLATE Hepatocyte Plating Media (SEKISUI MEDICAL CO., LTD., Tokyo, Japan) and cultured for 4 h at 37°C. The medium was replaced with OptiCULTURE Hepatocyte Culture Media (SEKISUI MEDICAL) and cultured for 24 h at 37 or 39°C. Differentiated HepaRG cells were purchased from KAC Co., Ltd., and the cells were thawed in HepaRG medium 670 (KAC Co., Ltd. Tokyo, Japan). HepaRG cells were seeded on 96-well plates with HepaRG medium 670 and cultured for 4 h at 37°C. Then, the medium was replaced, and the cells were cultured for 24 h at 37°C or 39°C. ﻿HepG2 cells were cultured with high-glucose DMEM (Sigma-Aldrich) supplemented with 10% (v/v) fetal bovine serum (Cell Culture Bioscience, Tokyo, Japan), 1% (v/v) penicillin/streptomycin (Wako), and 1% (v/v) MEM Non-essential Amino Acids Solution (Wako). HepG2 cells were seeded on 96-well plates with the medium and cultured for 24 h at 37°C. After cell attachment, HepG2 cells were cultured for 24 h or 12 days at 37°C or 39°C.

**Immunocytochemistry.** Cells were fixed with 4% paraformaldehyde (Wako, 161-20141) in PBS for 20 min at 25°C and then permeabilized with 0.1% Triton X-100 (MP Biomedicals, CA, USA) in PBS for 10 min at 25°C. Subsequently, cells were blocked in blocking buffer (5% normal goat serum, Vector; 5% normal donkey serum, Wako; 3% bovine serum albumin, Sigma-Aldrich; and 0.1% Tween-20, Nacalai Tesque, Inc., Kyoto, Japan) in PBS at 4°C for 16 h and then incubated at 4°C for 16 h with the primary antibody (anti-human CYP3A7 rabbit IgG, 1:500, Proteintech, Chicago, USA; anti-human A1AT rabbit IgG, 1:800, Dako, Tokyo, Japan. A0012; anti-human CYP3A4 mouse IgG, 1:25, Santa Cruz Biotechnology, Inc., CA, USA, sc-53850; and anti-human ALB mouse IgG, 1:50, R&D Systems, 188835) in blocking buffer. Cells were then incubated at 37°C for 60 min with a secondary antibody (AlexaFluor 488 Donkey anti-rabbit IgG, 1:1000, Jackson ImmunoResearch, PA, USA 711-546-152 and AlexaFluor 647 Donkey anti-mouse IgG, 1:1000, Jackson ImmunoResearch, 715-606-150) in blocking buffer before a final incubation with 4’,6-diamidino-2-phenylindole (DAPI; Wako 342-07431) at 25°C for 30 min.

**Indocyanine green (ICG) uptake/excretion assay.** Briefly, 1 mg mL^−1^ ICG (Sigma-Aldrich) was dissolved in the hepatocyte-maturation medium. Cells were treated with the ICG solution for 1 h, rinsed with hepatocyte-maturation medium, and then observed using a bright-field microscope (Olympus, Tokyo, Japan). After 24 h, the cells were observed again to visualize excretion capability. To calculate the ICG positive area, we used ImageJ version 1.52m (1) software ﻿(National Institutes of Health, Bethesda, MD, USA).

**Image acquisition.** Each sample containing cells was placed on the stage of a Nikon ECLIPSE Ti inverted fluorescence microscope equipped with a CFI plan fluor 10×/0.30 N.A. objective lens (Nikon, Tokyo, Japan), a CCD camera (ORCA-R2; Hamamatsu Photonics, Hamamatsu City, Japan), a mercury lamp (Intensilight; Nikon), an XYZ automated stage (Ti-S-ER motorized stage with encoders; Nikon), and filter cubes for fluorescence channels (DAPI and GFP HYQ; Nikon). For image acquisition, the exposure times were set at 200 ms for DAPI, 200 ms for GFP HYQ for A1AT and CYP3A7, and 800 ms for CYP3A4 and ALB.

**Periodic Acid Schiff (PAS) staining.** A PAS staining kit (Merck, Tokyo, Japan) was used following the manufacture’s protocol. To calculate the PAS-stained cell area, ImageJ 1.52m software was used.

**RNA purification.** RNA was purified from cells using the RNeasy Mini Kit (Qiagen, Hilden, Germany). The cells were directly lysed by adding 350 µL of lysis buffer in the kit and transferred to a 1.5-mL tube. Subsequently, 350 µL of 70% (v/v) ethanol was added to the tubes. Each solution was transferred to an RNeasy Mini spin column placed in a 2-mL collection tube. The column was centrifuged for 15 s at 8000 × g, and the flow-through was discarded. Then, 350 µL of buffer RW1 was added to the columns and centrifuged. Then, 80 µL of DNase digestion buffer was added to the column and incubated at 25°C for 15 min. Next, 350 µL of buffer RW1 was added to the column tube and centrifuged again. The column was washed with 500 µL of buffer RPE three times, placed in a new 2-mL tube, and centrifuged. The column was placed in a new 1.5-mL collection tube, and 50 µL of RNase-free water was added to the column; this was followed by centrifugation for 1 min at 8000× g to elute RNA into the collection tube. The RNA quality was evaluated using Agilent 2100 Bioanalyser (Agilent Technologies, Inc., USA).

**Quantitative PCR.** The primer sets used in this study are shown in **Table S3**. One µg of total RNA from each sample was used for cDNA synthesis using the PrimeScript RT Master Mix (TaKaRa). Each PCR was carried out in a final volume of 25 μL containing 12.5 μL of 2× TB Green Premix Ex Taq II (Tli RNaseH Plus), 2 μL (0.8 μM) of forward and reverse primers, 0.5 μL of ROX Reference Dye or Dye, 7.6 μL of nuclease-free water, and 0.4 μL of (50 ng/µl) of cDNA template. The qPCR cycling conditions were 95°C for 2 min, 40 cycles at 95°C for 15 s, and 60°C for 1 min. The relative mRNA expression of some genes was calculated using the *ΔΔ*Ct method, and housekeeping genes were used to normalize the transcript levels.

**mRNA-seq analysis.** Sequenced reads were processed and demultiplexed using EPI2ME software (Oxford NANOPORE Technologies). Failed reads were discarded, and Fast5 files were converted into FASTQ and FASTA files using EPI2ME software. The generated FASTQ files were loaded to BioJupies (2) for alignment with human genomes and annotation. The expressed genes were counted using BioJupies (**Table S2**), and the counts were analyzed using TCC (3) in R using the Bioconductor package. Briefly, the counted dataset was introduced into the TCC-GUI package. Gene counts were normalized using TMM (Trimmed mean of M values) methods (4) in the edgeR package (5) with the following parameters: Filtering Threshold for Low Count Genes 30, Number of Iterations = 3; FDR < 0.1; Elimination of Potential DEGs = 0.05. Time-course RNA sequence analysis was conducted according to the R package microarray Significant Profiles (maSigPro) (version 1.60.0) (6) using normalized expression value. GO analysis for DEGs was performed using the WEB-based Gene Set Analysis Toolkit (WebGestalt (7)). “Biological Process noRedundant,” “Cellular component noRedundant,” and “molecular function noRedundant” were selected for the database, and “genome protein-coding” genes were selected for the reference set. Additionally, “KEGG” and “Reactome” were selected for the database. The protein-coding genes among the DEGs served as inputs.

**Data availability (RNA-seq)**. The mRNA-seq data have been deposited in the NCBI Gene Expression Omnibus under accession number GSE172227.

**Statistical analysis.** All experiments were carried out at least three times independently. Statistical analysis was performed with Microsoft excel. The results for the CYP3A4 assay and albumin secretion assay are presented as means ± SD. In others, where applicable, center lines of box plots indicate medians; box limits indicate the 25th and 75th percentiles as determined by R software; whiskers extend 1.5 times the interquartile range from the 25th and 75th percentiles. ﻿Comparisons between the two groups were conducted using a two-tailed Student’s *t*-test. ﻿Statistical significance was accepted for values of p < 0.05 in the present study. Comparison of CYP3A7, CYP3A4, ALB, A1AT, AFP, OATP1B3, NTCP, COL1A, COL3A, and COL4A positive cells between two groups was conducted using the two-sample Kolmogorov–Smirnov test followed by the Wilcoxon rank-sum test.

**
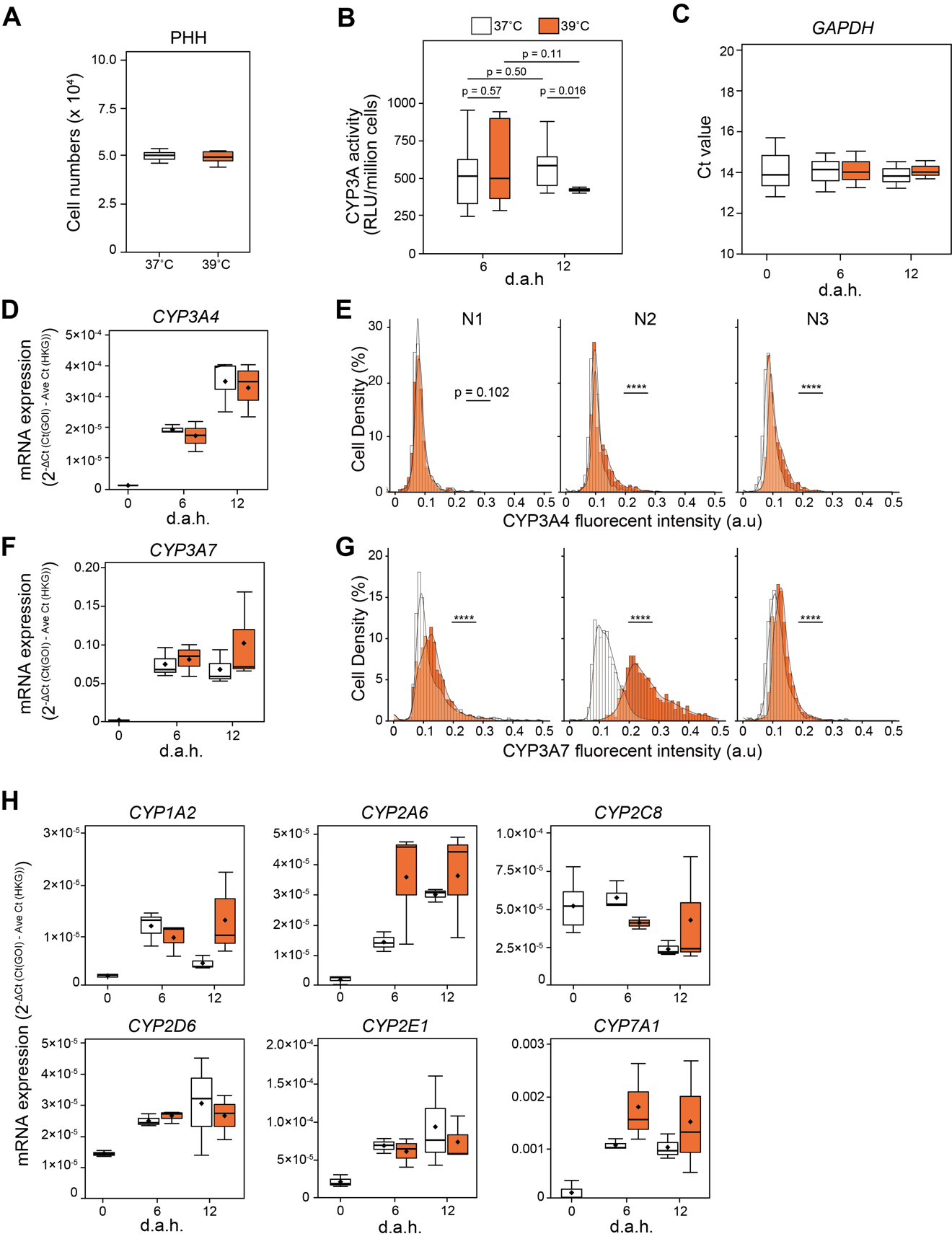
**

**Fig. S1. A.** Box plots showing the cell numbers of living primary human hepatocytes (PHH) cultured at 39°C for a day (n = 4). **B.** Bioluminescent-based CYP3A activity assay to confirm CYP3A activities of HepG2 cells cultured at 39°C for 12 d. **C.** Estimation of cycle threshold (Ct) values of glyceraldehyde-3-phosphate dehydrogenase (*GAPDH*) during hepatic differentiation with heat treatments at 37°C and 39°C. **D.** Quantification of mRNA expression of cytochrome p450 3A4 (*CYP3A4*) during hepatic differentiation with heat treatments at 37°C and 39°C. **E.** Microscopic single-cell profiling of CYP3A4 in 37°C- and hESC-HLCs treated at 39°C at 12 d.a.h. with three biological replicates. **F.** Quantification of mRNA expression of cytochrome p450 3A7 (*CYP3A7*) during hepatic differentiation with heat treatments at 37°C and 39°C. **G.** Microscopic single-cell profiling of CYP3A7 in 37°C- and 39°C-treated hESC-HLCs at 12 d.a.h. with three biological replicates. **H.** Quantification of mRNA expression of typical hepatic CYP enzymes, *CYP1A2*, *CYP2A6*, *CYP2C8*, *CYP2D6*, *CYP2E1*, *CYP3A4*, *CYP3A7*, and *CYP7A1* during hepatic differentiation with heat treatments at 37°C and 39°C. All experiments were conducted in three biological replicates (n = 3). Centerlines of box plots indicate medians; box limits indicate the 25^th^ and 75^th^ percentiles as determined using the *R* software; whiskers extend 1.5 times the interquartile range from the 25^th^ and 75^th^ percentiles.

**
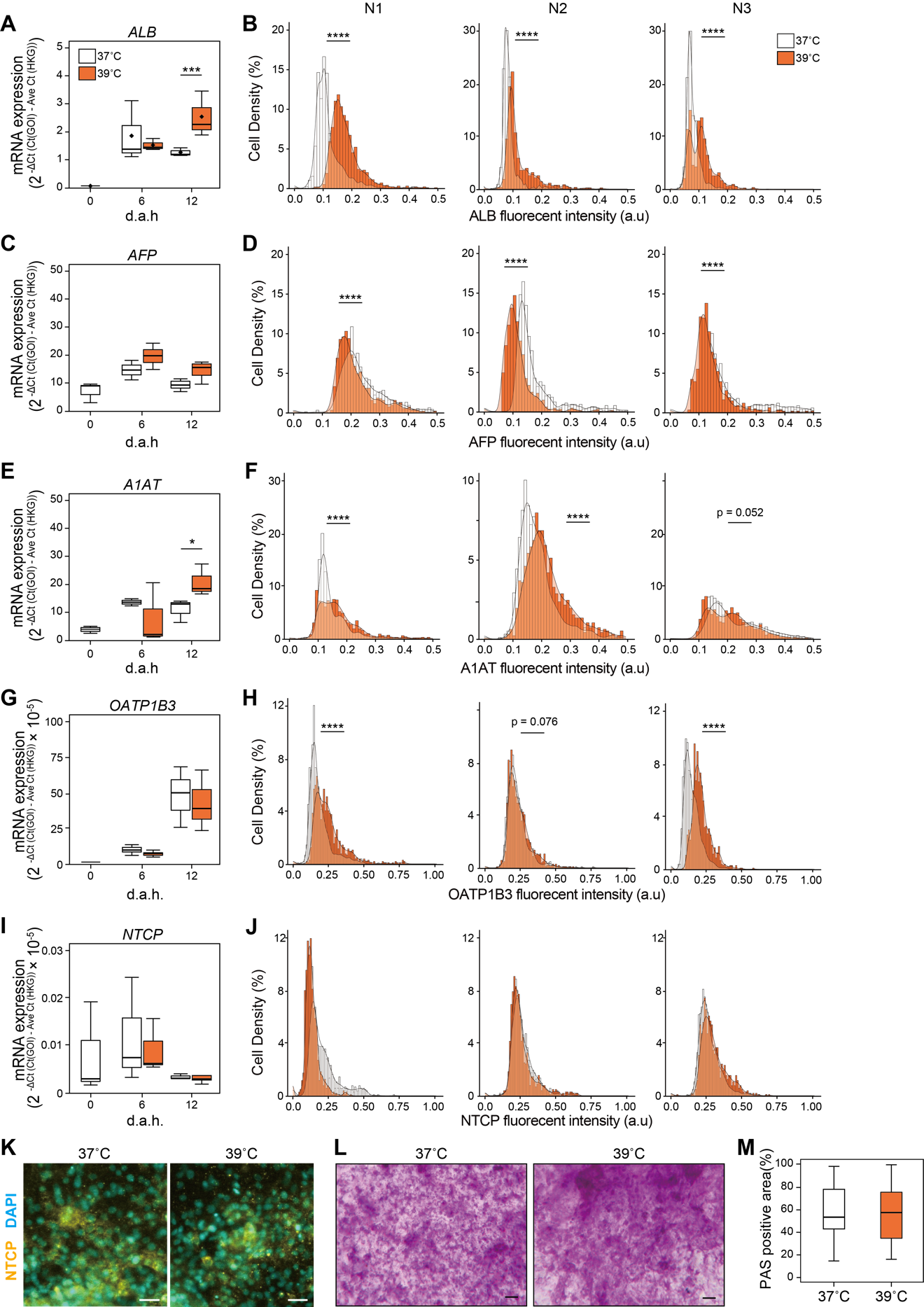
**

**Fig. S2** **A.** Gene expression analysis of albumin (ALB) during hepatic differentiation with heat treatments at 37°C and 39°C. **B.** Single-cell profiling of ALB in 37°C- and 39°C-treated hESC-HLCs at 12 d.a.h. with three biological replicates. **C.** Gene expression analysis of α-fetoprotein (AFP) during hepatic differentiation with heat treatments at 37°C and 39°C. **D.** Single-cell profiling of AFP in 37°C- and 39°C-treated hESC-HLCs at 12 d.a.h. with three biological replicates. **E.** Gene expression analysis of α1-anti trypsin (A1AT) during hepatic differentiation with heat treatments at 37°C and 39°C. **F.** Single-cell profiling of A1AT in 37°C- and 39°C-treated hESC-HLCs at 12 d.a.h. with three biological replicates. **G.** Gene expression analysis of organic anion transporting polypeptide 1B3 (OATP1B3) during hepatic differentiation with heat treatments at 37°C and 39°C. **H.** Single-cell profiling of OATP1B3 in 37°C- and 39°C-treated hESC-HLCs at 12 d.a.h. with three biological replicates. **I.** Gene expression analysis of Na^+^-taurocholate cotransporting polypeptide (*NCTP* or known as sodium/bile acid cotransporter) during hepatic differentiation with heat treatments at 37°C and 39°C. **J.** Single-cell profiling of NTCP in 37°C- and 39°C-treated hESC-HLCs at 12 d.a.h. with three biological replicates. **K.** Immunofluorescent micrographs of NTCP in 37°C- and 39°C-treated hESC-HLCs at 12 d.a.h. DAPI was used for nuclei staining. **L, M**, Micrographs (**L**) and the percentiles of positive area (**M**) of 37°C- and 39°C-treated hESC-HLCs at 12 d.a.h., stained with periodic acid schiff (PAS). Scale bars = 50 µm. (n = 20). All experiments were conducted in three biological replicates (n = 3)

**
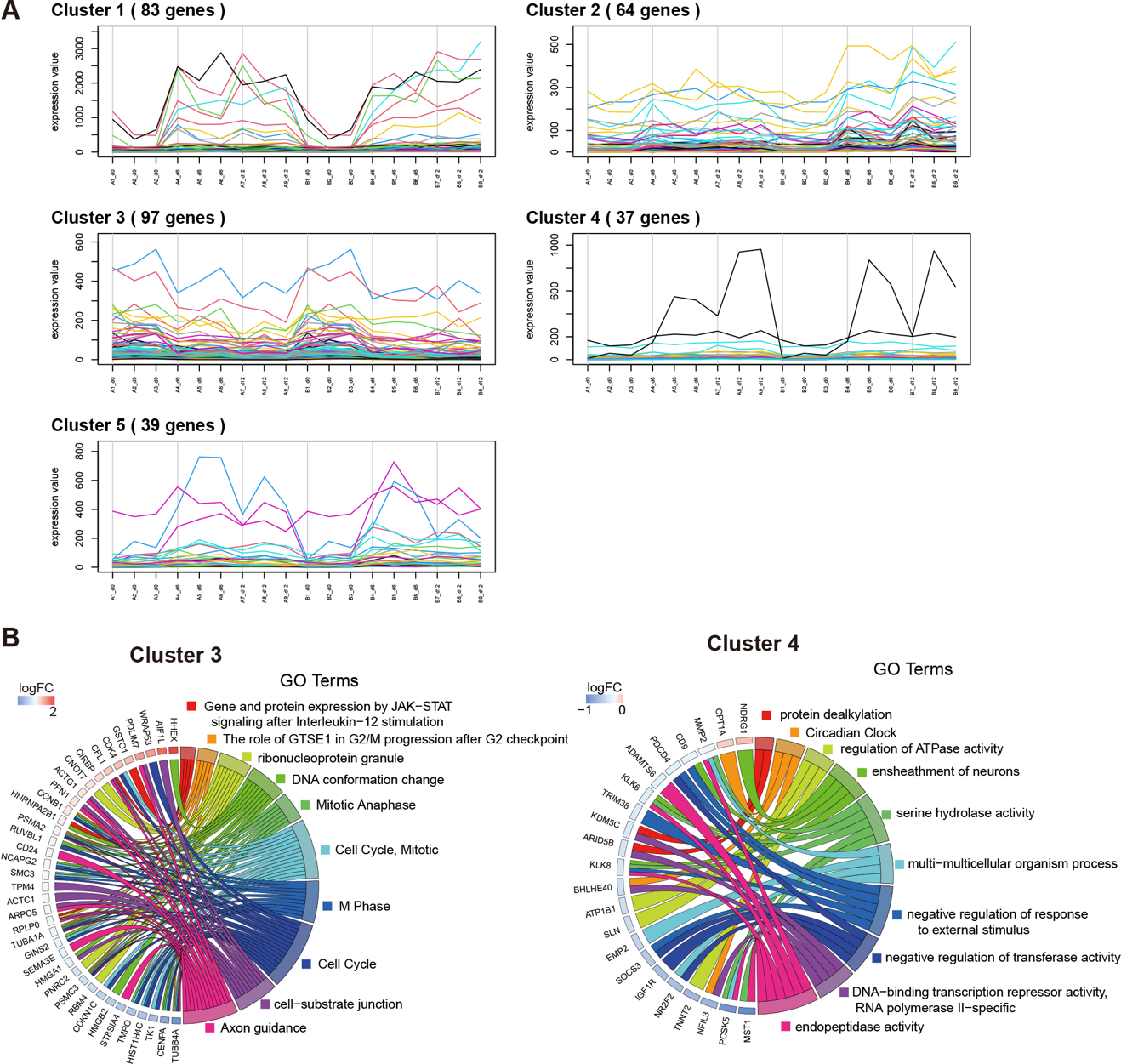
**

**Fig. S3 A.** Five clusters of specific gene signatures in hESC-HLCs with heat treatments at 37°C and 39°C by global transcriptional analysis to identify. **B.** Chord diagram presenting enriched GO clusters of the differentially expressed genes of hESC-HLCs with heat treatment at 37°C and 39°C. In each chord diagram, enriched GO clusters are shown (right), and genes contributing to this enrichment shown (left). Each cluster is shown in Figure 4A.

**
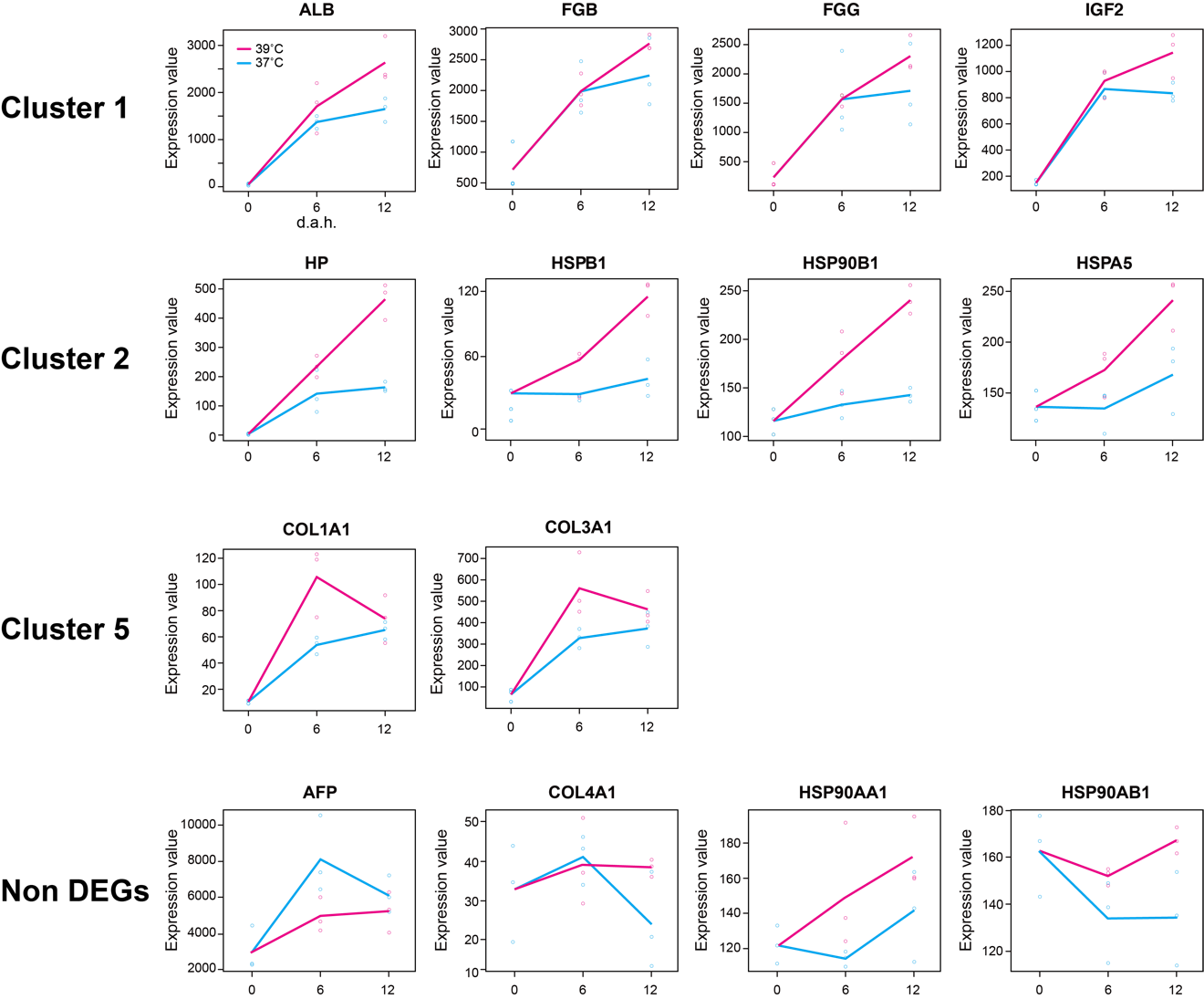
**

**Fig. S4** Time-course plots of typical genes in clusters 1, 2, and 5, and non DEGs.

**
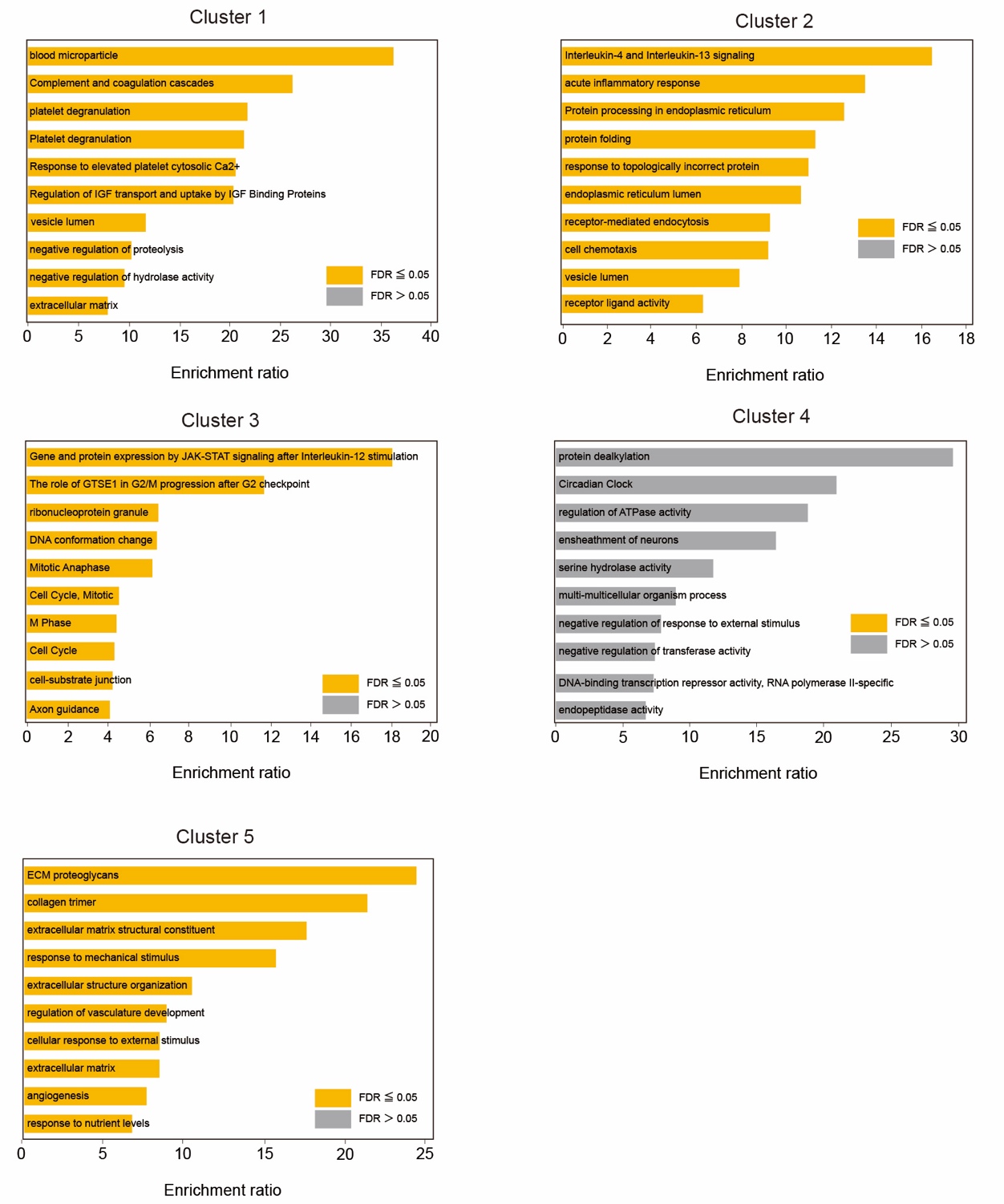
**

**Fig. S5** Enriched GO, KEGG, and Reactome terms of differentially expressed genes in 39°C-treated hESC-HLCs.

**
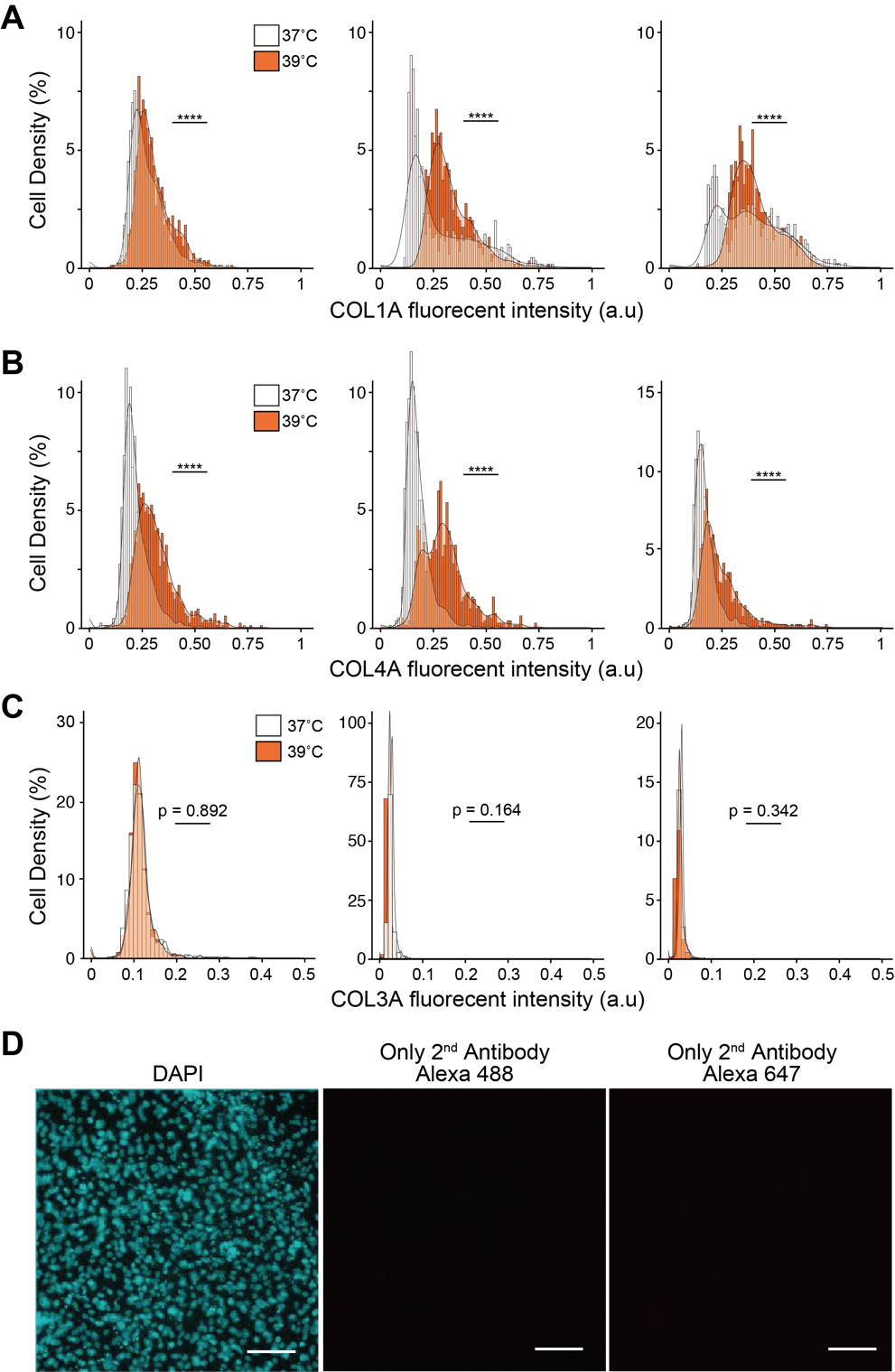
**

**Fig. S6** **A-C.** Single-cell profiling of collagen type I (COL1A) (**A**), collagen type IV (COL4A) (**B**), and collagen type III (COL3A) (**C**) in 37°C- and 39°C-treated hESC-HLCs at 12 d.a.h. using three biological replicates. **D.** The negative immunochemistry control stained with only 2^nd^ antibody labeled with Alexa 488 and 647 fluorescent dyes.

**Table S1.** List of differentially expressed genes (DEGs) between 37°C- and 39°C-treated hESC-HLCs.

**Table S2.** List of gene ontological terms obtained with DEGs between 37°C- and 39°C-treated hESC-HLCs.

**Table S3.** List of primers for quantitative RT-PCR in this study.

**References**

1. Schneider CA, Rasband WS, Eliceiri KW. NIH Image to ImageJ: 25 years of image analysis. Nat. Methods [Internet]. 2012;9:671–675. Available from: https://doi.org/10.1038/nmeth.2089

2. Torre D, Lachmann A, Ma’ayan A. BioJupies: Automated Generation of Interactive Notebooks for RNA-Seq Data Analysis in the Cloud. Cell Syst. [Internet]. 2018;7:556-561.e3. Available from: https://doi.org/10.1016/j.cels.2018.10.007

3. Su W, Sun J, Shimizu K, Kadota K. TCC-GUI: a Shiny-based application for differential expression analysis of RNA-Seq count data. BMC Res. Notes [Internet]. 2019;12:133. Available from: https://doi.org/10.1186/s13104-019-4179-2

4. Robinson MD, Oshlack A. A scaling normalization method for differential expression analysis of RNA-seq data. Genome Biol. [Internet]. 2010;11:R25. Available from: https://doi.org/10.1186/gb-2010-11-3-r25

5. Robinson MD, McCarthy DJ, Smyth GK. edgeR: a Bioconductor package for differential expression analysis of digital gene expression data. Bioinformatics [Internet]. 2010;26:139–140. Available from: https://doi.org/10.1093/bioinformatics/btp616

6. Conesa A, Nueda MJ, Ferrer A, Talon M. maSigPro: a method to identify significantly differential expression profiles in time-course microarray experiments. Bioinformatics [Internet]. 2006;22:1096–1102. Available from: https://doi.org/10.1093/bioinformatics/btl056

7. Wang J, Vasaikar S, Shi Z, Greer M, Zhang B. WebGestalt 2017: a more comprehensive, powerful, flexible and interactive gene set enrichment analysis toolkit. Nucleic Acids Res. [Internet]. 2017;45:W130–W137. Available from: https://doi.org/10.1093/nar/gkx356

Supporting Table S1

| **gene** | **p-value** | **R-squared** | **p.valor_beta0** | **p.valor_Group.BvsGroup.A** | **p.valor_Time** | **p.valor_TimexGroup.B** | **p.valor_Time2** | **p.valor_Time2xGroup.B** |
| --- | --- | --- | --- | --- | --- | --- | --- | --- |
| AADAC | 3.78E-10 | 0.769894 | NA | NA | 0.001889 | 0.00219 | NA | 0.006676 |
| ACKR3 | 7.51E-11 | 0.751739 | 0.032495 | NA | 0.000945 | NA | 0.000435 | 0.002046 |
| ACSL4 | 1.86E-12 | 0.810671 | 1.80E-08 | NA | 0.000213 | 0.010453 | NA | 0.03493 |
| ACTC1 | 4.19E-26 | 0.876888 | 1.57E-17 | NA | 6.09E-06 | NA | 0.000576 | NA |
| ACTG1 | 2.84E-13 | 0.813342 | 1.79E-22 | NA | 0.000348 | 0.016201 | 0.027072 | NA |
| ADAMTS6 | 2.16E-16 | 0.824313 | 3.96E-08 | NA | 4.38E-06 | 0.008722 | NA | 0.010212 |
| AGR3 | 1.26E-12 | 0.753435 | 0.026521 | NA | NA | 0.00308 | 0.029276 | 0.012456 |
| AGT | 1.97E-25 | 0.887016 | 1.88E-11 | NA | 3.26E-07 | NA | 2.37E-05 | NA |
| AHCY | 5.47E-13 | 0.774041 | 1.10E-17 | NA | 0.000738 | NA | 0.031458 | NA |
| AHSG | 5.83E-09 | 0.730045 | 2.37E-11 | NA | 0.000191 | NA | 0.004729 | NA |
| AIF1L | 3.78E-14 | 0.805109 | 3.81E-10 | NA | 0.000199 | 0.03464 | NA | 0.019075 |
| AKR1C1 | 1.03E-10 | 0.776936 | NA | NA | 0.003158 | NA | 0.01341 | 0.008615 |
| ALB | 5.29E-91 | 0.962406 | 8.26E-15 | NA | 9.87E-11 | 0.029649 | 1.36E-08 | NA |
| ALDH7A1 | 3.47E-37 | 0.922338 | 1.93E-19 | NA | 1.14E-07 | 6.12E-06 | NA | 3.81E-06 |
| ALDOB | 1.57E-25 | 0.863978 | NA | NA | 2.59E-05 | 0.000177 | 1.78E-05 | NA |
| AMBP | 7.29E-33 | 0.894849 | 1.10E-16 | NA | 3.18E-07 | NA | 5.69E-05 | NA |
| ANG | 1.13E-20 | 0.848147 | 1.45E-13 | NA | 4.30E-06 | NA | 0.000267 | NA |
| ANGPTL3 | 1.03E-13 | 0.791611 | 0.010698 | NA | 0.000124 | NA | 0.002281 | NA |
| ANXA3 | 9.00E-09 | 0.713746 | 2.08E-22 | NA | 0.000416 | NA | 0.005552 | NA |
| APOC3 | 3.52E-14 | 0.804592 | 1.27E-11 | NA | 1.02E-05 | NA | 0.000683 | NA |
| APOC4-APOC2 | 5.00E-12 | 0.769147 | 1.44E-05 | NA | 6.96E-05 | NA | 0.002256 | NA |
| APOH | 1.10E-23 | 0.852548 | NA | NA | 3.11E-06 | 0.007388 | 6.75E-06 | NA |
| AREG | 2.57E-34 | 0.914332 | 1.61E-06 | NA | 0.008209 | 9.16E-06 | NA | 0.000493 |
| ARG1 | 1.55E-15 | 0.808676 | 0.007983 | NA | 0.000188 | 0.000674 | 0.000296 | NA |
| ARID5B | 1.48E-09 | 0.724665 | 3.58E-10 | NA | 0.000105 | 0.001658 | NA | 0.001466 |
| ARIH1 | 2.87E-14 | 0.830819 | 1.22E-14 | NA | NA | 0.000983 | 0.000127 | 0.002628 |
| ARL4A | 1.59E-09 | 0.730395 | 1.60E-05 | NA | 0.002011 | NA | 0.039276 | NA |
| ARPC5 | 3.29E-09 | 0.737872 | 1.02E-20 | NA | 0.001179 | 0.000647 | NA | 0.000985 |
| ASGR2 | 3.98E-13 | 0.798094 | 1.00E-07 | NA | 5.00E-06 | NA | 2.85E-05 | NA |
| ATG3 | 6.22E-07 | 0.726778 | 3.91E-14 | NA | 0.01032 | 0.001527 | 0.003516 | 0.000354 |
| ATP1B1 | 2.07E-13 | 0.816131 | 2.74E-24 | NA | 7.28E-06 | 0.001171 | NA | 0.000155 |
| B2M | 6.62E-50 | 0.94146 | 1.09E-22 | NA | 1.17E-06 | 2.74E-05 | 3.56E-05 | NA |
| BCHE | 8.53E-16 | 0.74459 | 6.12E-08 | NA | NA | NA | 0.000276 | NA |
| BDH2 | 3.44E-18 | 0.848742 | 4.29E-13 | NA | 1.60E-05 | 0.001702 | 1.09E-06 | NA |
| BEX1 | 1.77E-13 | 0.819472 | 5.07E-16 | NA | 0.000511 | 0.002438 | NA | 0.010604 |
| BEX5 | 1.17E-20 | 0.828843 | NA | NA | 3.49E-06 | 0.002873 | 2.81E-06 | NA |
| BHLHE40 | 1.59E-10 | 0.713491 | NA | NA | 9.37E-05 | NA | 0.000457 | NA |
| C10orf10 | 1.45E-13 | 0.794794 | 4.44E-13 | NA | 7.13E-05 | NA | 0.001229 | NA |
| C15orf48 | 8.86E-20 | 0.825084 | 0.001994 | NA | 0.000117 | 0.000694 | 0.000513 | NA |
| C3 | 5.29E-42 | 0.928162 | NA | NA | 8.37E-06 | 0.002764 | 3.21E-05 | NA |
| C5orf45 | 1.96E-07 | 0.707087 | 0.019421 | NA | 0.000245 | 0.011508 | NA | 0.010181 |
| CALD1 | 1.24E-09 | 0.729592 | 3.86E-23 | NA | 0.000439 | NA | 0.007676 | NA |
| CALU | 2.52E-32 | 0.913269 | 7.78E-25 | NA | 0.006673 | 7.85E-08 | 0.009107 | NA |
| CANX | 1.92E-10 | 0.774375 | 3.01E-22 | NA | NA | 0.004122 | 0.021998 | 0.037752 |
| CAV1 | 2.57E-22 | 0.875535 | 5.22E-06 | NA | 9.74E-05 | 6.06E-05 | 0.00022 | NA |
| CCL20 | 1.14E-14 | 0.809792 | 7.93E-06 | NA | 0.020041 | 3.06E-05 | NA | NA |
| CCNB1 | 3.39E-17 | 0.796408 | 1.45E-18 | NA | 3.41E-07 | NA | NA | NA |
| CCT6A | 4.79E-09 | 0.725357 | 5.63E-19 | NA | 0.00081 | NA | 0.013534 | NA |
| CD24 | 7.81E-12 | 0.796717 | 2.99E-23 | NA | 3.44E-05 | 0.003283 | NA | 0.00227 |
| CD9 | 2.56E-13 | 0.813378 | 8.86E-18 | NA | 3.46E-05 | 0.001056 | NA | 0.001383 |
| CDK4 | 3.72E-09 | 0.752801 | 1.73E-16 | NA | NA | 0.004481 | 3.40E-05 | 0.002019 |
| CDKN1A | 1.31E-08 | 0.732632 | 2.46E-05 | NA | 0.006415 | 0.035898 | 0.04115 | NA |
| CDKN1C | 8.05E-11 | 0.788516 | 1.00E-16 | NA | 0.000134 | 0.001699 | 7.90E-05 | NA |
| CENPA | 2.14E-12 | 0.790748 | 3.53E-12 | NA | 0.002963 | 0.00167 | 0.011116 | NA |
| CFB | 4.87E-32 | 0.896272 | 1.71E-06 | NA | 2.58E-07 | NA | 1.28E-05 | NA |
| CFH | 5.17E-16 | 0.775267 | NA | NA | 0.003441 | 0.036599 | 0.004756 | NA |
| CFHR1 | 1.77E-09 | 0.721551 | NA | NA | 0.014812 | 0.002581 | 0.007852 | NA |
| CFI | 6.22E-19 | 0.856272 | NA | NA | 6.79E-05 | 0.000617 | 8.43E-05 | NA |
| CFL1 | 1.92E-08 | 0.747035 | 1.12E-18 | NA | NA | 0.001195 | 6.32E-05 | 0.000583 |
| CHI3L1 | 4.20E-15 | 0.764663 | NA | NA | 3.15E-05 | NA | 6.43E-05 | NA |
| CHST12 | 1.58E-09 | 0.76284 | 0.001345 | NA | 0.000246 | NA | 0.000751 | 0.013855 |
| CIRBP | 3.74E-13 | 0.840174 | 3.69E-14 | NA | 0.012126 | 0.002518 | 0.000558 | 0.002843 |
| CLDN18 | 7.03E-11 | 0.76375 | NA | NA | 0.000326 | 0.008999 | 0.005496 | NA |
| CNN3 | 1.82E-45 | 0.943862 | 8.97E-24 | NA | 2.79E-07 | 0.046286 | 4.59E-06 | 0.012403 |
| CNOT7 | 4.75E-13 | 0.809398 | 6.84E-22 | NA | 0.00011 | NA | 0.007543 | 0.001037 |
| COL14A1 | 2.76E-16 | 0.779978 | NA | NA | 0.000276 | NA | 0.000909 | NA |
| COL17A1 | 1.09E-10 | 0.742182 | 0.04969 | NA | 0.002417 | NA | 0.00111 | 0.00703 |
| COL1A1 | 1.92E-85 | 0.966415 | 7.58E-13 | NA | 1.83E-07 | 0.000399 | 2.89E-05 | 0.001158 |
| COL3A1 | 8.38E-22 | 0.85461 | 1.21E-14 | NA | 4.83E-07 | NA | 2.41E-05 | NA |
| COL5A1 | 2.43E-15 | 0.794782 | NA | NA | 1.43E-05 | NA | 9.96E-05 | NA |
| COMMD8 | 4.62E-11 | 0.75354 | 6.68E-19 | NA | NA | 6.57E-06 | NA | 1.46E-05 |
| COMMD9 | 4.39E-09 | 0.722209 | 8.10E-18 | NA | NA | NA | 2.34E-05 | 0.011444 |
| COMT | 2.52E-26 | 0.894981 | 1.80E-14 | NA | 0.000154 | 0.00035 | NA | 0.006306 |
| CPA2 | 7.25E-46 | 0.92359 | NA | NA | 4.76E-07 | NA | 1.08E-06 | 0.032195 |
| CPSF3 | 1.80E-08 | 0.734933 | 6.81E-14 | NA | NA | 0.005813 | 8.14E-05 | 0.001657 |
| CPT1A | 5.73E-21 | 0.83529 | NA | NA | 0.001659 | NA | 0.004859 | NA |
| CRLS1 | 3.28E-09 | 0.731785 | 2.63E-09 | NA | NA | 0.004888 | 0.000531 | 0.000801 |
| CST3 | 1.54E-09 | 0.768453 | 8.38E-17 | NA | 0.013358 | 0.000201 | 0.007969 | NA |
| CST4 | 8.74E-18 | 0.817322 | 0.048625 | NA | 0.004555 | NA | 0.018304 | NA |
| CTSV | 3.88E-71 | 0.958137 | 1.05E-18 | NA | 9.35E-07 | 0.018555 | 0.000782 | NA |
| CXCL12 | 1.81E-19 | 0.869627 | 0.000498 | NA | 0.000154 | 5.12E-05 | 9.95E-05 | NA |
| CXCL17 | 1.92E-09 | 0.741898 | 8.28E-06 | NA | 0.001418 | NA | 0.03154 | NA |
| CXCL5 | 1.32E-11 | 0.719971 | NA | NA | NA | 1.18E-05 | NA | NA |
| CYB5A | 8.54E-19 | 0.856572 | 1.16E-19 | NA | 2.90E-05 | NA | 0.000618 | 0.015382 |
| CYSTM1 | 4.18E-13 | 0.815049 | 7.93E-17 | NA | 0.007367 | 7.70E-05 | 0.008289 | NA |
| DAZAP2 | 1.84E-08 | 0.702576 | 2.46E-10 | NA | 0.001144 | 0.040192 | 0.00521 | NA |
| DCN | 1.23E-10 | 0.762048 | 1.05E-10 | NA | 8.72E-06 | NA | 2.96E-05 | NA |
| DDIT3 | 1.15E-16 | 0.839587 | 2.10E-14 | NA | 0.001237 | NA | 0.009838 | 0.000547 |
| DEFB1 | 8.37E-11 | 0.7075 | NA | NA | 9.54E-05 | NA | 0.000546 | NA |
| DEPTOR | 1.66E-09 | 0.750041 | 3.47E-12 | NA | NA | 7.64E-05 | 0.009977 | 7.86E-05 |
| DHRS7 | 8.00E-39 | 0.925965 | 7.55E-18 | NA | 8.69E-06 | 0.027734 | 0.001823 | NA |
| DIO3 | 1.70E-13 | 0.787841 | 3.15E-14 | NA | 2.89E-06 | NA | NA | NA |
| DPPA3 | 2.01E-14 | 0.779722 | 1.01E-09 | NA | 0.001698 | NA | 0.042829 | NA |
| DPYSL3 | 6.76E-25 | 0.894669 | 5.18E-16 | NA | 4.31E-07 | NA | 2.16E-05 | 0.000845 |
| EDARADD | 4.53E-15 | 0.827431 | 9.73E-14 | NA | 0.002404 | 0.000623 | NA | NA |
| EDNRA | 7.80E-12 | 0.772462 | 3.34E-09 | NA | 0.000993 | 0.001866 | 0.001112 | NA |
| EIF4EBP2 | 4.37E-15 | 0.832058 | 5.46E-19 | NA | 4.64E-05 | 0.020223 | 0.002754 | NA |
| EMP2 | 6.29E-11 | 0.771188 | 4.21E-17 | NA | 1.57E-05 | NA | 0.00019 | 0.040925 |
| EMP3 | 1.42E-10 | 0.768086 | 1.42E-07 | NA | NA | 0.000412 | 0.018729 | 0.003396 |
| ENO1 | 8.96E-10 | 0.737203 | 1.10E-24 | NA | NA | NA | 7.55E-06 | 0.000281 |
| ERBB4 | 2.41E-09 | 0.714006 | 2.82E-10 | NA | 0.002738 | NA | 0.042329 | NA |
| ERCC1 | 1.57E-08 | 0.70805 | 1.39E-15 | NA | NA | 0.006118 | 0.011563 | NA |
| ERO1A | 5.78E-32 | 0.907815 | 1.51E-12 | NA | 0.001746 | 1.23E-06 | 0.004915 | NA |
| ERP44 | 5.94E-08 | 0.710671 | 8.95E-21 | NA | 0.007833 | NA | 0.03462 | 0.00026 |
| FABP1 | 5.86E-15 | 0.818871 | 7.45E-11 | NA | 9.95E-06 | NA | 0.00067 | NA |
| FAM20B | 6.50E-07 | 0.70583 | 1.04E-05 | NA | 0.0392 | NA | 0.003589 | 0.007749 |
| FAM96A | 9.91E-13 | 0.778715 | 7.04E-18 | NA | 0.000177 | NA | 0.006876 | NA |
| FCGRT | 2.60E-12 | 0.767433 | 0.008171 | NA | 3.91E-05 | NA | 0.000335 | NA |
| FGB | 1.02E-10 | 0.767245 | 3.33E-18 | NA | 0.000629 | NA | 0.024378 | NA |
| FGG | 1.10E-10 | 0.762478 | 9.17E-14 | NA | 0.000141 | NA | 0.005912 | NA |
| FGL1 | 6.18E-14 | 0.784119 | NA | NA | 0.000117 | NA | 0.000514 | NA |
| FOS | 4.11E-09 | 0.72322 | 7.29E-05 | NA | 0.001534 | NA | 0.025347 | NA |
| FXYD3 | 7.97E-16 | 0.828837 | NA | NA | 0.000661 | 0.000521 | 0.000425 | NA |
| FXYD5 | 2.35E-29 | 0.908295 | 3.30E-09 | NA | 0.007916 | 3.67E-05 | NA | 0.001699 |
| G0S2 | 8.78E-12 | 0.759179 | NA | NA | 9.29E-05 | NA | 0.00026 | NA |
| GADD45A | 3.51E-07 | 0.705354 | 7.86E-09 | NA | 0.000315 | NA | 0.000348 | 0.010357 |
| GALNT2 | 2.68E-09 | 0.722644 | 0.001159 | NA | 0.004513 | NA | 0.00158 | 0.000401 |
| GC | 3.28E-51 | 0.936238 | NA | NA | 1.00E-05 | 0.00189 | 1.14E-05 | NA |
| GDF15 | 3.29E-15 | 0.745533 | 5.15E-07 | NA | 0.046297 | NA | NA | 1.09E-05 |
| GINS2 | 1.02E-26 | 0.87557 | 7.44E-16 | NA | 2.23E-08 | NA | NA | NA |
| GLT8D1 | 1.98E-10 | 0.76739 | 2.96E-06 | NA | 3.96E-05 | NA | 1.45E-05 | 0.003678 |
| GPI | 4.24E-17 | 0.846864 | 5.19E-18 | NA | NA | 0.037861 | 0.000701 | 0.000515 |
| GPRC5A | 9.89E-08 | 0.707311 | 8.28E-06 | NA | 0.003462 | 0.027574 | 0.012459 | NA |
| GPS1 | 5.07E-09 | 0.747933 | 2.30E-13 | NA | NA | 0.006755 | 0.000101 | 0.003441 |
| GPX7 | 5.42E-08 | 0.732114 | 1.95E-08 | NA | 0.000124 | NA | 0.0002 | 0.026287 |
| GSTK1 | 1.16E-10 | 0.749547 | 2.65E-18 | NA | 0.000803 | NA | 0.024963 | NA |
| GSTO1 | 4.12E-12 | 0.768477 | 7.54E-21 | NA | 2.63E-06 | NA | NA | 0.000223 |
| GUSB | 5.56E-11 | 0.75266 | 1.00E-08 | NA | 9.28E-06 | NA | 3.40E-05 | NA |
| HACD1 | 2.10E-13 | 0.787187 | 2.37E-15 | NA | 2.33E-05 | NA | 0.000307 | NA |
| HADHB | 1.29E-16 | 0.84277 | 3.03E-14 | NA | 0.000239 | NA | 4.41E-05 | 4.59E-06 |
| HAMP | 9.08E-20 | 0.811468 | 0.013549 | NA | 2.80E-05 | NA | 0.000812 | NA |
| HGD | 7.06E-16 | 0.800252 | NA | NA | 0.00106 | NA | 0.001271 | NA |
| HHEX | 2.19E-14 | 0.78875 | 3.74E-09 | NA | 5.98E-05 | 0.044018 | NA | 0.007324 |
| HIGD1A | 2.31E-07 | 0.711328 | 1.40E-17 | NA | 0.035523 | NA | 0.007563 | 0.000115 |
| HIST1H4C | 3.01E-16 | 0.830046 | 3.17E-21 | NA | 0.009869 | NA | NA | 7.79E-05 |
| HIST1H4I | 7.35E-13 | 0.822143 | 0.001643 | NA | 0.000232 | 0.001136 | NA | 0.003668 |
| HMGA1 | 4.11E-23 | 0.885461 | 2.23E-17 | NA | 3.01E-06 | 0.005648 | NA | 0.01235 |
| HMGB2 | 2.32E-29 | 0.896035 | 1.08E-20 | NA | 4.63E-05 | 3.52E-05 | NA | NA |
| HMGCL | 2.37E-09 | 0.72184 | 1.51E-13 | NA | 2.48E-05 | NA | 0.000109 | NA |
| HNRNPA2B1 | 2.20E-14 | 0.814973 | 2.10E-24 | NA | 1.35E-06 | 0.022774 | NA | NA |
| HP | 8.22E-85 | 0.962238 | 1.01E-05 | NA | 2.55E-09 | 0.000168 | 6.66E-08 | NA |
| HPX | 9.55E-08 | 0.703964 | 1.71E-14 | NA | 0.00088 | NA | 0.016123 | NA |
| HSD3B1 | 5.75E-33 | 0.897214 | NA | NA | 1.48E-06 | NA | 1.58E-06 | 0.003276 |
| HSP90B1 | 2.96E-25 | 0.883496 | 9.32E-24 | NA | 0.016 | 4.59E-06 | NA | NA |
| HSPA5 | 2.50E-11 | 0.754717 | 1.94E-23 | NA | NA | 0.000935 | 0.024038 | NA |
| HSPB1 | 2.33E-49 | 0.936412 | 4.66E-22 | NA | NA | 4.59E-08 | 0.004733 | NA |
| ID1 | 4.47E-10 | 0.741869 | 9.64E-15 | NA | 0.002625 | 0.000144 | NA | 0.000205 |
| ID3 | 2.99E-15 | 0.796314 | 2.87E-17 | NA | 1.79E-05 | NA | 0.000361 | NA |
| IER3IP1 | 7.79E-09 | 0.746428 | 4.74E-18 | NA | 0.000755 | 0.008376 | NA | 0.017711 |
| IFI16 | 1.57E-15 | 0.795472 | NA | NA | 0.000564 | 0.0237 | 0.001052 | NA |
| IFITM1 | 1.19E-13 | 0.794615 | 3.07E-10 | NA | 0.008576 | 0.001125 | NA | 0.011744 |
| IFITM3 | 7.63E-13 | 0.798454 | 2.20E-17 | NA | 0.000614 | 0.000507 | NA | 0.001892 |
| IGF1R | 7.63E-14 | 0.787082 | 2.65E-14 | NA | 1.37E-06 | NA | NA | 0.004424 |
| IGF2 | 2.25E-165 | 0.982008 | 1.50E-21 | NA | 3.56E-12 | NA | 4.98E-10 | 0.004152 |
| ITGB6 | 1.86E-10 | 0.728205 | NA | NA | 0.000584 | 0.013709 | 0.000743 | NA |
| ITIH1 | 5.57E-26 | 0.879654 | 3.37E-07 | NA | 1.25E-06 | NA | 5.56E-05 | NA |
| KDM5C | 1.07E-13 | 0.819799 | 9.49E-17 | NA | NA | 8.29E-05 | 1.38E-05 | 8.62E-05 |
| KLB | 6.79E-10 | 0.719212 | 1.51E-06 | NA | 1.88E-05 | NA | NA | NA |
| KLK6 | 4.53E-20 | 0.852495 | 5.29E-06 | NA | 1.86E-06 | NA | 3.04E-05 | NA |
| KLK8 | 2.88E-09 | 0.735337 | NA | NA | 0.000272 | NA | 0.002015 | NA |
| KNG1 | 1.82E-14 | 0.829945 | 2.66E-05 | NA | 2.42E-05 | 0.010734 | 0.000322 | 0.024819 |
| LAPTM4A | 7.98E-10 | 0.764632 | 1.59E-24 | NA | 0.000181 | NA | 0.000643 | 0.011802 |
| LBP | 2.43E-73 | 0.962243 | 0.001266 | NA | 1.46E-09 | NA | 8.65E-09 | 0.000568 |
| LDHA | 7.48E-09 | 0.727969 | 1.41E-14 | NA | 0.004114 | 0.001766 | 0.006548 | NA |
| LEAP2 | 2.42E-15 | 0.809471 | NA | NA | 0.000732 | 0.006296 | 0.004401 | NA |
| LIPC | 4.92E-11 | 0.807692 | 4.25E-08 | NA | 1.71E-05 | 0.006214 | 7.65E-05 | 0.006964 |
| LMAN1 | 2.96E-10 | 0.746159 | 4.23E-19 | NA | 0.001979 | 0.021702 | NA | NA |
| LRRC75A | 1.44E-08 | 0.74039 | 3.51E-22 | NA | 0.000468 | NA | 0.00099 | 0.005113 |
| LSM12 | 1.53E-07 | 0.71044 | 5.03E-15 | NA | NA | 0.045038 | 5.84E-05 | 0.008892 |
| LUM | 3.07E-09 | 0.743411 | 5.02E-17 | NA | 0.000329 | 0.005713 | 0.000333 | NA |
| LUZP1 | 9.53E-10 | 0.728172 | 6.76E-16 | NA | 0.000106 | NA | 0.001054 | NA |
| MANBA | 6.18E-08 | 0.740915 | 4.15E-14 | NA | 0.003879 | 0.001216 | 0.026056 | 0.00187 |
| MED12L | 1.29E-12 | 0.755845 | 4.19E-16 | NA | NA | 5.82E-06 | NA | NA |
| MMP2 | 2.58E-14 | 0.791526 | 0.004043 | NA | 2.05E-05 | NA | 0.000223 | NA |
| MST1 | 4.71E-13 | 0.777623 | NA | NA | 0.000365 | NA | 0.004657 | 0.008762 |
| MT-ATP6 | 2.68E-12 | 0.759388 | 5.32E-19 | NA | 2.30E-06 | NA | NA | NA |
| MT-CO1 | 2.71E-15 | 0.816403 | 1.51E-22 | NA | 3.33E-05 | NA | 0.001409 | NA |
| MT-CO3 | 1.45E-21 | 0.880284 | 1.24E-20 | NA | 2.69E-05 | 0.007871 | 0.000475 | NA |
| MT-CYB | 4.54E-15 | 0.814388 | 1.25E-23 | NA | 0.001362 | 0.001557 | NA | NA |
| MT-ND4 | 2.85E-10 | 0.725907 | 2.27E-18 | NA | 8.68E-06 | NA | NA | NA |
| MT-ND5 | 5.36E-18 | 0.839968 | 1.60E-20 | NA | 6.84E-05 | 0.007053 | NA | NA |
| MT-ND6 | 1.61E-12 | 0.749293 | 1.61E-14 | NA | 2.76E-06 | NA | NA | NA |
| MTPAP | 3.33E-10 | 0.744952 | 1.15E-12 | NA | 0.000104 | NA | NA | 3.33E-05 |
| MTRNR2L1 | 2.21E-10 | 0.753396 | 6.57E-22 | NA | 0.000223 | NA | 0.004986 | NA |
| MYO9A | 5.06E-12 | 0.790081 | 1.61E-15 | NA | 0.000216 | NA | 0.000254 | 0.000273 |
| NCAPG2 | 7.90E-11 | 0.701217 | 1.93E-09 | NA | 2.51E-05 | NA | NA | NA |
| NDRG1 | 5.75E-21 | 0.871086 | 1.47E-05 | NA | 3.39E-05 | 0.045477 | 0.000649 | NA |
| NFIL3 | 1.91E-10 | 0.774623 | NA | NA | NA | 0.0164 | 1.08E-05 | 0.007936 |
| NID1 | 1.76E-12 | 0.760279 | 2.47E-11 | NA | 7.73E-05 | 0.001613 | NA | 0.000281 |
| NNMT | 9.78E-15 | 0.832106 | NA | NA | 0.001065 | 0.001968 | 0.002902 | NA |
| NQO1 | 8.74E-32 | 0.909849 | 1.76E-18 | NA | 3.24E-07 | NA | 1.58E-05 | NA |
| NR2F2 | 8.44E-11 | 0.770929 | 1.71E-12 | NA | 3.11E-05 | NA | 0.000482 | 0.011977 |
| NREP | 2.57E-10 | 0.738973 | 1.16E-15 | NA | 0.000809 | NA | 0.017971 | NA |
| NRP2 | 5.73E-09 | 0.743222 | NA | NA | 0.008752 | 0.013847 | 0.016521 | NA |
| NTS | 4.43E-10 | 0.841403 | 2.52E-08 | NA | 0.000639 | NA | NA | NA |
| OLFML3 | 4.08E-52 | 0.94837 | 1.69E-14 | NA | 8.34E-10 | NA | 2.91E-09 | 0.000576 |
| ORM2 | 6.98E-29 | 0.877014 | NA | NA | 1.61E-06 | NA | 3.31E-05 | NA |
| OS9 | 8.88E-07 | 0.73029 | 1.34E-12 | NA | 0.000121 | 0.002772 | 0.000121 | 0.001512 |
| P4HA1 | 1.59E-18 | 0.844062 | 2.84E-21 | NA | NA | NA | 1.79E-07 | NA |
| P4HA2 | 1.64E-08 | 0.705535 | NA | NA | 0.002455 | 0.019153 | 0.005996 | NA |
| P4HB | 3.86E-10 | 0.787255 | 3.29E-19 | NA | 0.000518 | 0.002313 | 6.61E-05 | 0.000376 |
| PCSK5 | 2.66E-09 | 0.749898 | 1.07E-08 | NA | 4.40E-05 | 0.003396 | NA | 0.000636 |
| PDCD4 | 2.81E-09 | 0.721701 | 7.36E-15 | NA | 0.000438 | NA | 0.008464 | NA |
| PDE4DIP | 2.59E-09 | 0.71511 | 0.033918 | NA | 0.000222 | NA | 0.001797 | NA |
| PDGFRA | 8.41E-15 | 0.775365 | 1.15E-15 | NA | 1.04E-06 | NA | NA | NA |
| PDLIM7 | 4.44E-12 | 0.76078 | 2.44E-09 | NA | NA | 0.046907 | 0.001162 | 0.003168 |
| PEX2 | 6.34E-14 | 0.823847 | 2.46E-11 | NA | 7.03E-06 | 0.027047 | 3.35E-06 | NA |
| PFN1 | 7.73E-10 | 0.767945 | 3.41E-20 | NA | 3.54E-05 | 0.024105 | NA | 0.010829 |
| PGD | 3.97E-11 | 0.757358 | 3.70E-17 | NA | 0.000103 | NA | 0.001602 | NA |
| PHACTR2 | 1.70E-27 | 0.900252 | 8.68E-20 | NA | 1.50E-05 | 2.01E-05 | NA | 8.22E-05 |
| PLA2G2A | 1.67E-74 | 0.953714 | 1.82E-05 | NA | 4.72E-06 | 0.000586 | 0.000295 | 0.011189 |
| PLD3 | 1.93E-08 | 0.72236 | 2.48E-17 | NA | 0.000603 | 0.001352 | NA | 0.001155 |
| PLG | 5.26E-21 | 0.854237 | 0.00026 | NA | 9.14E-07 | NA | 8.60E-06 | NA |
| PLIN2 | 2.17E-39 | 0.923802 | 7.77E-17 | NA | 0.000245 | 0.000319 | 0.031416 | NA |
| PLP2 | 6.27E-31 | 0.901563 | NA | NA | 0.000163 | 8.96E-06 | 0.000316 | NA |
| PLTP | 4.27E-11 | 0.763024 | 3.49E-16 | NA | 1.78E-05 | NA | 6.66E-06 | NA |
| PNRC2 | 2.31E-08 | 0.723004 | 1.54E-18 | NA | 0.001214 | 0.003557 | 0.001455 | NA |
| POLR2E | 2.91E-14 | 0.838845 | 1.89E-17 | NA | 0.029162 | 6.18E-05 | 0.000885 | 2.79E-05 |
| PON2 | 6.68E-21 | 0.881375 | 9.28E-15 | NA | 0.000501 | 0.04187 | 0.002842 | 0.006142 |
| POSTN | 6.68E-14 | 0.800069 | NA | NA | 0.009174 | 0.000102 | NA | 0.000448 |
| PPP1R3C | 4.46E-11 | 0.782776 | NA | NA | 0.004149 | 0.011783 | 0.005529 | NA |
| PRR15L | 1.98E-09 | 0.718159 | NA | NA | 0.000858 | NA | 0.001425 | 0.010177 |
| PRSS23 | 2.81E-10 | 0.77106 | 1.65E-11 | NA | 0.017625 | 0.000639 | 0.034224 | NA |
| PSMA2 | 1.02E-09 | 0.705274 | 1.76E-26 | NA | 1.65E-05 | NA | NA | NA |
| PSMC3 | 1.50E-09 | 0.722852 | 8.19E-18 | NA | 0.002216 | NA | NA | 0.031019 |
| PTN | 5.69E-12 | 0.770638 | 1.35E-14 | NA | 6.51E-06 | 0.023473 | 7.73E-05 | NA |
| PTRHD1 | 1.47E-09 | 0.725891 | 7.33E-17 | NA | 0.00268 | NA | NA | 0.024644 |
| RBFA | 1.15E-08 | 0.705458 | 2.08E-07 | NA | NA | 0.030515 | 0.002862 | 0.031745 |
| RBM3 | 9.70E-14 | 0.733197 | 9.31E-15 | NA | NA | NA | 2.56E-06 | NA |
| RBM4 | 8.21E-10 | 0.731818 | 2.59E-20 | NA | NA | 0.001135 | NA | 0.017504 |
| RBP2 | 1.60E-30 | 0.886439 | 0.002976 | NA | 3.75E-07 | 0.039258 | 2.33E-06 | NA |
| RGN | 1.00E-10 | 0.755111 | 4.78E-06 | NA | 2.35E-05 | NA | 0.000139 | NA |
| ROCK1 | 2.05E-12 | 0.776492 | 0.001718 | NA | 6.61E-05 | NA | 0.000914 | NA |
| RPLP0 | 2.78E-08 | 0.731739 | 2.00E-23 | NA | 0.000414 | 0.015962 | NA | 0.020373 |
| RPS27L | 1.42E-11 | 0.785037 | 4.26E-17 | NA | 0.001439 | 0.004532 | 0.007475 | NA |
| RRBP1 | 4.11E-07 | 0.741135 | 5.46E-18 | NA | 0.000331 | 0.004979 | 0.001805 | 0.007394 |
| RRP7A | 2.56E-09 | 0.714819 | 4.69E-10 | NA | 9.06E-05 | NA | NA | 0.000985 |
| RTN4 | 1.72E-18 | 0.82188 | 1.45E-22 | NA | NA | 2.51E-07 | NA | NA |
| RUVBL1 | 1.52E-13 | 0.819675 | 2.97E-15 | NA | 3.25E-05 | 0.008024 | NA | 0.007968 |
| S100A3 | 2.49E-08 | 0.738015 | NA | NA | 0.009687 | 0.001893 | NA | 0.007801 |
| S100A4 | 1.68E-26 | 0.890325 | 2.50E-16 | NA | 6.84E-07 | 0.000635 | 2.06E-06 | NA |
| SAA1 | 4.89E-09 | 0.756093 | NA | NA | 0.00541 | 0.008237 | NA | 0.027376 |
| SAA2 | 2.48E-15 | 0.771642 | NA | NA | NA | 3.12E-06 | NA | NA |
| SAA4 | 3.00E-32 | 0.912924 | NA | NA | 0.001548 | 0.000192 | NA | 0.002456 |
| SAT1 | 1.16E-12 | 0.80894 | 1.63E-23 | NA | 4.62E-05 | NA | 5.53E-06 | 0.000794 |
| SEC23A | 5.43E-08 | 0.722623 | 1.01E-16 | NA | NA | 0.015075 | 8.33E-05 | 0.011368 |
| SEMA3E | 9.73E-09 | 0.721552 | 1.37E-06 | NA | 0.00072 | NA | NA | NA |
| SEMA5A | 1.04E-10 | 0.766463 | 0.018569 | NA | 0.000274 | NA | 0.003376 | NA |
| SERINC2 | 3.27E-09 | 0.739601 | 2.01E-15 | NA | 0.001404 | 0.000781 | 0.000539 | NA |
| SERPINA1 | 1.45E-17 | 0.828754 | 1.37E-19 | NA | 3.22E-06 | NA | 0.000124 | NA |
| SERPINA3 | 7.28E-67 | 0.949211 | NA | NA | 1.00E-07 | 0.000113 | 3.80E-07 | NA |
| SERPINA4 | 7.82E-11 | 0.714571 | NA | NA | 0.000576 | NA | 0.003618 | NA |
| SERPINA6 | 3.64E-16 | 0.809442 | NA | NA | 1.88E-05 | NA | 0.00012 | NA |
| SERPINA7 | 2.23E-07 | 0.734972 | 0.002565 | NA | 0.000157 | 0.049748 | 0.000602 | NA |
| SERPINB6 | 2.08E-06 | 0.70064 | 4.27E-16 | NA | 0.000259 | 0.036761 | 0.0008 | 0.035606 |
| SERPINB9 | 1.67E-11 | 0.730837 | 1.75E-15 | NA | 5.79E-06 | NA | NA | NA |
| SERPIND1 | 1.78E-11 | 0.776408 | 0.000109 | NA | 1.40E-05 | NA | 6.41E-05 | NA |
| SERPINH1 | 2.31E-11 | 0.752027 | 1.31E-19 | NA | 9.67E-05 | NA | 0.001605 | NA |
| SFRP2 | 4.36E-14 | 0.789618 | NA | NA | 0.000489 | NA | 0.000822 | NA |
| SIL1 | 1.92E-08 | 0.725963 | 8.95E-09 | NA | 0.0011 | NA | 0.005381 | 0.025268 |
| SLC1A3 | 2.96E-19 | 0.84098 | 1.20E-07 | NA | 1.04E-06 | NA | 1.16E-05 | NA |
| SLC25A6 | 2.58E-14 | 0.778635 | 4.74E-25 | NA | NA | NA | 8.53E-07 | NA |
| SLC35F2 | 8.00E-12 | 0.759974 | 2.63E-13 | NA | 2.96E-05 | NA | 0.000451 | NA |
| SLC38A3 | 7.63E-09 | 0.708085 | NA | NA | 0.001279 | NA | 0.005249 | NA |
| SLC7A8 | 1.89E-12 | 0.816163 | 1.12E-13 | NA | 3.05E-05 | 0.038336 | NA | 0.022584 |
| SLN | 5.60E-16 | 0.772018 | 2.21E-10 | NA | 9.06E-06 | NA | 0.000726 | NA |
| SMARCA1 | 4.22E-11 | 0.733624 | 7.80E-17 | NA | 0.00027 | NA | 0.007937 | NA |
| SMC3 | 1.76E-18 | 0.845982 | 2.28E-19 | NA | 7.83E-06 | NA | 0.000233 | NA |
| SMDT1 | 1.65E-06 | 0.718105 | 7.10E-14 | NA | 0.000419 | 0.044156 | 0.000164 | 0.015033 |
| SOCS3 | 9.12E-08 | 0.712602 | 7.03E-14 | NA | NA | NA | 5.60E-05 | 0.048123 |
| SPARC | 2.27E-08 | 0.706601 | 3.73E-24 | NA | NA | 0.000634 | NA | 0.005714 |
| SPOCK2 | 4.76E-11 | 0.724562 | 4.11E-11 | NA | 0.000157 | 0.04572 | NA | 0.036683 |
| SSBP2 | 1.99E-08 | 0.720752 | 5.40E-08 | NA | 0.004402 | NA | 0.010683 | 0.00314 |
| SSR1 | 4.47E-09 | 0.71364 | 1.60E-21 | NA | NA | 0.002851 | NA | 0.000347 |
| ST8SIA4 | 3.74E-10 | 0.71986 | 7.86E-08 | NA | 0.003196 | NA | 0.01543 | NA |
| STMN1 | 1.35E-12 | 0.776382 | 5.36E-20 | NA | 0.003684 | NA | NA | 0.001763 |
| STRADB | 6.85E-10 | 0.74181 | 2.86E-10 | NA | NA | 0.018798 | 0.000655 | 0.00181 |
| SULF1 | 1.10E-17 | 0.830369 | NA | NA | 1.25E-05 | 0.011834 | 2.64E-05 | NA |
| TAC3 | 3.16E-10 | 0.756131 | 3.96E-12 | NA | NA | 0.043946 | 0.044267 | 0.007053 |
| TF | 5.14E-18 | 0.835547 | 7.48E-07 | NA | 1.17E-06 | NA | 2.30E-05 | NA |
| TFG | 1.84E-09 | 0.750896 | 6.25E-13 | NA | 0.006363 | 0.000211 | 0.002163 | NA |
| TGM2 | 2.58E-17 | 0.860508 | 2.18E-06 | NA | 0.000196 | 0.045638 | 0.004028 | NA |
| TIMP1 | 7.00E-28 | 0.899204 | 1.62E-12 | NA | 0.000118 | 1.49E-05 | NA | 0.000185 |
| TK1 | 6.66E-14 | 0.764645 | 2.24E-15 | NA | NA | 0.001278 | 0.001082 | NA |
| TLE1 | 2.08E-12 | 0.796952 | 4.05E-07 | NA | 0.00514 | 0.003241 | 0.030615 | NA |
| TM4SF4 | 1.24E-08 | 0.724595 | 8.17E-14 | NA | 0.004962 | 0.018449 | 0.02621 | NA |
| TM4SF5 | 4.40E-09 | 0.711952 | 0.023143 | NA | 0.000505 | NA | 0.004773 | NA |
| TMED10 | 1.93E-07 | 0.712061 | 7.37E-18 | NA | 0.006969 | 0.001762 | 0.006391 | NA |
| TMED3 | 3.00E-11 | 0.776124 | 1.96E-14 | NA | 0.001214 | NA | 0.00064 | 7.50E-05 |
| TMEM176A | 7.54E-15 | 0.822357 | NA | NA | 0.000107 | 0.023521 | 0.000276 | NA |
| TMEM176B | 7.54E-50 | 0.932546 | NA | NA | 5.18E-07 | 0.000813 | 1.21E-06 | NA |
| TMEM208 | 2.25E-09 | 0.718245 | 2.13E-14 | NA | 8.78E-05 | NA | 0.000951 | NA |
| TMEM59 | 8.94E-09 | 0.744706 | 5.76E-21 | NA | 0.003733 | NA | 0.012634 | 0.003838 |
| TMEM88 | 5.55E-12 | 0.731437 | 2.69E-11 | NA | 1.33E-05 | NA | NA | NA |
| TMPO | 1.05E-14 | 0.822292 | 1.30E-17 | NA | 0.007031 | NA | 0.042416 | 0.000233 |
| TMSB15B | 4.78E-14 | 0.798042 | 9.91E-13 | NA | 0.000576 | NA | 0.025535 | NA |
| TMSB4Y | 1.81E-10 | 0.782352 | 7.33E-10 | NA | 0.011794 | 5.76E-05 | NA | 0.000187 |
| TNNT2 | 5.32E-09 | 0.702976 | 0.047188 | NA | 5.92E-05 | NA | 0.000182 | NA |
| TPM3 | 2.51E-16 | 0.817375 | 2.45E-22 | NA | 4.88E-05 | NA | 0.002093 | NA |
| TPM4 | 1.14E-09 | 0.738333 | 3.13E-18 | NA | 0.000173 | NA | 0.002151 | NA |
| TRA2B | 9.93E-14 | 0.82496 | 9.48E-16 | NA | 0.000106 | 0.000221 | NA | 0.000205 |
| TRIM38 | 6.38E-11 | 0.71404 | 1.23E-12 | NA | NA | NA | 7.49E-06 | NA |
| TRPM6 | 3.79E-19 | 0.813095 | 0.005171 | NA | 4.41E-05 | 2.79E-05 | NA | 2.73E-05 |
| TSPAN8 | 3.49E-14 | 0.776977 | NA | NA | 0.000977 | 0.010891 | NA | NA |
| TTC39C | 4.65E-09 | 0.722825 | NA | NA | 0.000769 | NA | 0.001784 | 0.022936 |
| TUBA1A | 8.09E-11 | 0.76451 | 2.10E-15 | NA | 0.000296 | NA | 0.005581 | NA |
| TUBB4A | 3.66E-11 | 0.743126 | 7.49E-14 | NA | NA | NA | 0.032283 | 0.000748 |
| TXNIP | 2.17E-18 | 0.855091 | 0.0005 | NA | 7.37E-06 | 0.021603 | 2.62E-05 | NA |
| UBA5 | 7.42E-10 | 0.717698 | 1.63E-14 | NA | 0.002059 | 0.026379 | NA | NA |
| UBD | 1.37E-34 | 0.913248 | 1.61E-10 | NA | 8.58E-06 | 0.000131 | 0.000171 | NA |
| VAT1 | 4.62E-10 | 0.791894 | 6.86E-16 | NA | 0.006275 | 1.90E-05 | 0.001096 | 1.47E-05 |
| VCAN | 5.28E-18 | 0.810686 | 8.08E-20 | NA | 2.72E-07 | NA | NA | NA |
| VEZF1 | 8.31E-09 | 0.701977 | 3.78E-08 | NA | NA | NA | 0.000872 | 0.005789 |
| VIMP | 5.83E-07 | 0.735711 | 3.17E-14 | NA | 0.00011 | 0.007429 | 0.000282 | 0.006806 |
| VKORC1 | 3.59E-12 | 0.779083 | 4.15E-17 | NA | 0.000138 | NA | 0.004401 | NA |
| WARS | 2.16E-13 | 0.801488 | 3.57E-11 | NA | 2.31E-05 | NA | 0.00013 | 0.016439 |
| WDR77 | 1.69E-09 | 0.737472 | 1.03E-16 | NA | NA | NA | 2.22E-05 | 0.037262 |
| WRAP53 | 2.86E-10 | 0.729428 | 1.49E-06 | NA | NA | NA | 0.005804 | 0.007443 |
| YPEL5 | 5.80E-10 | 0.724794 | 3.03E-15 | NA | 0.000586 | NA | 0.014401 | NA |
| ZG16 | 1.59E-13 | 0.78884 | 0.000756 | NA | 6.20E-05 | NA | 0.001122 | NA |

**Table S2**

| Cluster | geneSet | description | Link | size | overlap | expect | enrichmentRatio | pValue | FDR | database | userId |
| --- | --- | --- | --- | --- | --- | --- | --- | --- | --- | --- | --- |
| 1 | GO:0072562 | blood microparticle | <http://amigo.geneontology.org/amigo/term/GO:0072562> | 108 | 17 | 0.46826448 | 36.3042695 | 0 | 0 | geneontology_Cellular_Component_noRedundant | AGT;AHSG;ALB;AMBP;C3;CFB;CFH;CFHR1;FGB;FGG;GC;HPX;ITIH1;KNG1;ORM2;PLG;TF |
| 1 | GO:0031983 | vesicle lumen | <http://amigo.geneontology.org/amigo/term/GO:0031983> | 337 | 17 | 1.46115862 | 11.6346027 | 5.54E-14 | 9.30E-11 | geneontology_Cellular_Component_noRedundant | AHSG;ALB;APOH;ARG1;C3;FGB;FGG;HPX;IGF2;KNG1;ORM2;PLG;ROCK1;SERPINA1;SERPINA4;TF;ZG16 |
| 1 | GO:0051346 | negative regulation of hydrolase activity | <http://amigo.geneontology.org/amigo/term/GO:0051346> | 431 | 18 | 1.86872215 | 9.63225058 | 2.30E-13 | 1.99E-10 | geneontology_Biological_Process_noRedundant | AGT;AHSG;AMBP;ANGPTL3;APOC3;C3;CNN3;CST4;FABP1;ITIH1;KNG1;RGN;ROCK1;SERPINA1;SERPINA4;SERPINA6;SERPINA7;SERPIND1 |
| 1 | GO:0002576 | platelet degranulation | <http://amigo.geneontology.org/amigo/term/GO:0002576> | 127 | 12 | 0.55064435 | 21.7926509 | 2.46E-13 | 1.99E-10 | geneontology_Biological_Process_noRedundant | AHSG;ALB;APOH;FGB;FGG;IGF2;KNG1;ORM2;PLG;SERPINA1;SERPINA4;TF |
| 1 | R-HSA-114608 | Platelet degranulation | <http://reactome.org/PathwayBrowser/#/R-HSA-114608> | 129 | 12 | 0.55931591 | 21.4547804 | 2.97E-13 | 1.99E-10 | pathway_Reactome | AHSG;ALB;APOH;FGB;FGG;IGF2;KNG1;ORM2;PLG;SERPINA1;SERPINA4;TF |
| 1 | R-HSA-76005 | Response to elevated platelet cytosolic Ca2+ | <http://reactome.org/PathwayBrowser/#/R-HSA-76005> | 134 | 12 | 0.58099482 | 20.6542289 | 4.70E-13 | 2.63E-10 | pathway_Reactome | AHSG;ALB;APOH;FGB;FGG;IGF2;KNG1;ORM2;PLG;SERPINA1;SERPINA4;TF |
| 1 | R-HSA-381426 | Regulation of Insulin-like Growth Factor (IGF) transport and uptake by Insulin-like Growth Factor Binding Proteins (IGFBPs) | <http://reactome.org/PathwayBrowser/#/R-HSA-381426> | 125 | 11 | 0.54197278 | 20.2962222 | 5.76E-12 | 2.77E-09 | pathway_Reactome | AHSG;ALB;C3;FGG;IGF2;KNG1;P4HB;PLG;SERPINA1;SERPIND1;TF |
| 1 | GO:0045861 | negative regulation of proteolysis | <http://amigo.geneontology.org/amigo/term/GO:0045861> | 336 | 15 | 1.45682284 | 10.296379 | 1.16E-11 | 4.89E-09 | geneontology_Biological_Process_noRedundant | AGT;AHSG;AMBP;C3;CST4;FABP1;ITIH1;KNG1;OS9;ROCK1;SERPINA1;SERPINA4;SERPINA6;SERPINA7;SERPIND1 |
| 1 | GO:0031012 | extracellular matrix | <http://amigo.geneontology.org/amigo/term/GO:0031012> | 496 | 17 | 2.15054799 | 7.90496192 | 2.72E-11 | 1.01E-08 | geneontology_Cellular_Component_noRedundant | AHSG;AMBP;ANG;APOC3;APOH;COL14A1;COL17A1;FGB;FGG;HPX;ITIH1;KNG1;LMAN1;ORM2;PLG;SERPINA1;ZG16 |
| 1 | hsa04610 | Complement and coagulation cascades | <http://www.kegg.jp/kegg-bin/show_pathway?hsa04610+2244+2266+3053+3075+3827+5265+5340+629+718> | 79 | 9 | 0.3425268 | 26.2753165 | 5.22E-11 | 1.75E-08 | pathway_KEGG | C3;CFB;CFH;FGB;FGG;KNG1;PLG;SERPINA1;SERPIND1 |
| 2 | GO:0005788 | endoplasmic reticulum lumen | <http://amigo.geneontology.org/amigo/term/GO:0005788> | 306 | 11 | 1.03191617 | 10.6597806 | 4.95E-09 | 1.66E-05 | geneontology_Cellular_Component_noRedundant | B2M;CALU;CANX;COL5A1;ERO1A;ERP44;HSP90B1;HSPA5;PRSS23;SIL1;TIMP1 |
| 2 | GO:0006898 | receptor-mediated endocytosis | <http://amigo.geneontology.org/amigo/term/GO:0006898> | 287 | 9 | 0.96784295 | 9.29902937 | 4.51E-07 | 4.05E-04 | geneontology_Biological_Process_noRedundant | ACKR3;B2M;CANX;CAV1;CFI;HP;HSP90B1;SAA1;SPARC |
| 2 | GO:0060326 | cell chemotaxis | <http://amigo.geneontology.org/amigo/term/GO:0060326> | 289 | 9 | 0.9745875 | 9.23467622 | 4.79E-07 | 2.00E+00 | geneontology_Biological_Process_noRedundant | ACKR3;CCL20;CXCL12;CXCL5;HSPB1;LBP;SAA1;SAA2;SAA4 |
| 2 | GO:0006457 | protein folding | <http://amigo.geneontology.org/amigo/term/GO:0006457> | 210 | 8 | 0.70817777 | 11.2965986 | 4.83E-07 | 4.05E-04 | geneontology_Biological_Process_noRedundant | B2M;CANX;ERO1A;ERP44;HSP90B1;HSPA5;HSPB1;SIL1 |
| 2 | GO:0002526 | acute inflammatory response | <http://amigo.geneontology.org/amigo/term/GO:0002526> | 154 | 7 | 0.51933036 | 13.4788961 | 8.15E-07 | 5.48E-04 | geneontology_Biological_Process_noRedundant | CFI;HP;LBP;SAA1;SAA2;SAA4;SERPINA3 |
| 2 | hsa04141 | Protein processing in endoplasmic reticulum | <http://www.kegg.jp/keggbin/show_pathway?hsa04141+1649+30001+3309+64374+6745+7184+821> | 165 | 7 | 0.55642539 | 12.580303 | 1.30E-06 | 7.21E-04 | pathway_KEGG | CANX;DDIT3;ERO1A;HSP90B1;HSPA5;SIL1;SSR1 |
| 2 | R-HSA-6785807 | Interleukin-4 and Interleukin-13 signaling | <http://reactome.org/PathwayBrowser/#/R-HSA-6785807> | 108 | 6 | 0.36420571 | 16.4742063 | 1.64E-06 | 7.21E-04 | pathway_Reactome | CDKN1A;FOS;HSP90B1;LBP;SAA1;TIMP1 |
| 2 | GO:0031983 | vesicle lumen | <http://amigo.geneontology.org/amigo/term/GO:0031983> | 337 | 9 | 1.1364567 | 7.91935142 | 1.72E-06 | 7.21E-04 | geneontology_Cellular_Component_noRedundant | B2M;ERP44;GPI;HP;HSP90B1;SAA1;SERPINA3;SPARC;TIMP1 |
| 2 | GO:0035966 | response to topologically incorrect protein | <http://amigo.geneontology.org/amigo/term/GO:0035966> | 188 | 7 | 0.63398772 | 11.0412234 | 3.09E-06 | 0.00107387 | geneontology_Biological_Process_noRedundant | DDIT3;ERO1A;ERP44;HSP90B1;HSPA5;HSPB1;SSR1 |
| 2 | GO:0048018 | receptor ligand activity | <http://amigo.geneontology.org/amigo/term/GO:0048018> | 468 | 10 | 1.57822474 | 6.33623321 | 3.20E-06 | 0.00107387 | geneontology_Molecular_Function_noRedundant | AREG;CCL20;CXCL12;CXCL5;GDF15;GPI;SAA1;SAA2;SAA4;TIMP1 |
| 3 | R-HSA-1640170 | Cell Cycle | <http://reactome.org/PathwayBrowser/#/R-HSA-1640170> | 641 | 16 | 3.62844755 | 4.40959936 | 5.62E-07 | 0.00188637 | pathway_Reactome | CCNB1;CDK4;CDKN1C;CENPA;GINS2;HIST1H4C;NCAPG2;PSMA2;PSMC3;RUVBL1;SMC3;TK1;TMPO;TUBA1A;TUBB4A;WRAP53 |
| 3 | R-HSA-69278 | Cell Cycle, Mitotic | <http://reactome.org/PathwayBrowser/#/R-HSA-69278> | 536 | 14 | 3.03408407 | 4.61424262 | 1.83E-06 | 0.00307094 | pathway_Reactome | CCNB1;CDK4;CDKN1C;CENPA;GINS2;HIST1H4C;NCAPG2;PSMA2;PSMC3;SMC3;TK1;TMPO;TUBA1A;TUBB4A |
| 3 | GO:0071103 | DNA conformation change | <http://amigo.geneontology.org/amigo/term/GO:0071103> | 245 | 9 | 1.38684813 | 6.48953539 | 1.02E-05 | 0.01107683 | geneontology_Biological_Process_noRedundant | CCNB1;CENPA;GINS2;HHEX;HMGA1;HMGB2;HNRNPA2B1;NCAPG2;RUVBL1 |
| 3 | R-HSA-422475 | Axon guidance | <http://reactome.org/PathwayBrowser/#/R-HSA-422475> | 551 | 13 | 3.11899314 | 4.16801174 | 1.32E-05 | 0.01107683 | pathway_Reactome | ACTG1;ARPC5;CD24;CFL1;PDLIM7;PFN1;PSMA2;PSMC3;RPLP0;SEMA3E;ST8SIA4;TUBA1A;TUBB4A |
| 3 | GO:0035770 | ribonucleoprotein granule | <http://amigo.geneontology.org/amigo/term/GO:0035770> | 214 | 8 | 1.21136938 | 6.60409624 | 2.87E-05 | 0.01929358 | geneontology_Cellular_Component_noRedundant | CIRBP;CNOT7;PNRC2;PSMA2;PSMC3;RBM4;RPLP0;TUBA1A |
| 3 | R-HSA-8852276 | The role of GTSE1 in G2/M progression after G2 checkpoint | <http://reactome.org/PathwayBrowser/#/R-HSA-8852276> | 75 | 5 | 0.42454535 | 11.777305 | 6.59E-05 | 0.03181218 | pathway_Reactome | CCNB1;PSMA2;PSMC3;TUBA1A;TUBB4A |
| 3 | R-HSA-8950505 | Gene and protein expression by JAK-STAT signaling after Interleukin-12 stimulation | <http://reactome.org/PathwayBrowser/#/R-HSA-8950505> | 39 | 4 | 0.22076358 | 18.1189307 | 6.80E-05 | 0.03181218 | pathway_Reactome | CFL1;GSTO1;HNRNPA2B1;RPLP0 |
| 3 | R-HSA-68886 | M Phase | <http://reactome.org/PathwayBrowser/#/R-HSA-68886> | 393 | 10 | 2.22461761 | 4.49515457 | 7.58E-05 | 0.03181218 | pathway_Reactome | CCNB1;CENPA;HIST1H4C;NCAPG2;PSMA2;PSMC3;SMC3;TMPO;TUBA1A;TUBB4A |
| 3 | GO:0030055 | cell-substrate junction | <http://amigo.geneontology.org/amigo/term/GO:0030055> | 411 | 10 | 2.32650849 | 4.29828648 | 1.10E-04 | 0.04092185 | geneontology_Cellular_Component_noRedundant | ACTC1;ACTG1;AIF1L;ARPC5;CFL1;HMGA1;PDLIM7;PFN1;RPLP0;TPM4 |
| 3 | R-HSA-68882 | Mitotic Anaphase | <http://reactome.org/PathwayBrowser/#/R-HSA-68882> | 199 | 7 | 1.12646032 | 6.21415589 | 1.36E-04 | 0.0426811 | pathway_Reactome | CENPA;PSMA2;PSMC3;SMC3;TMPO;TUBA1A;TUBB4A |
| 4 | GO:0017171 | serine hydrolase activity | <http://amigo.geneontology.org/amigo/term/GO:0017171> | 208 | 5 | 0.42587017 | 11.7406674 | 6.08E-05 | 0.16531355 | geneontology_Molecular_Function_noRedundant | KLK6;KLK8;MMP2;MST1;PCSK5 |
| 4 | GO:0007272 | ensheathment of neurons | <http://amigo.geneontology.org/amigo/term/GO:0007272> | 119 | 4 | 0.24364687 | 16.4172022 | 9.85E-05 | 0.16531355 | geneontology_Biological_Process_noRedundant | CD9;KLK6;KLK8;NDRG1 |
| 4 | GO:0004175 | endopeptidase activity | <http://amigo.geneontology.org/amigo/term/GO:0004175> | 436 | 6 | 0.89268939 | 6.72126282 | 2.28E-04 | 0.25525082 | geneontology_Molecular_Function_noRedundant | ADAMTS6;KLK6;KLK8;MMP2;MST1;PCSK5 |
| 4 | R-HSA-400253 | Circadian Clock | <http://reactome.org/PathwayBrowser/#/R-HSA-400253> | 70 | 3 | 0.14332169 | 20.9319328 | 3.91E-04 | 0.27089798 | pathway_Reactome | BHLHE40;CPT1A;NFIL3 |
| 4 | GO:0032102 | negative regulation of response to external stimulus | <http://amigo.geneontology.org/amigo/term/GO:0032102> | 312 | 5 | 0.63880525 | 7.82711161 | 4.03E-04 | 0.27089798 | geneontology_Biological_Process_noRedundant | CD9;KLK8;PDCD4;SOCS3;TRIM38 |
| 4 | GO:0043462 | regulation of ATPase activity | <http://amigo.geneontology.org/amigo/term/GO:0043462> | 78 | 3 | 0.15970131 | 18.7850679 | 5.37E-04 | 0.30063364 | geneontology_Biological_Process_noRedundant | ATP1B1;SLN;TNNT2 |
| 4 | GO:0044706 | multi-multicellular organism process | <http://amigo.geneontology.org/amigo/term/GO:0044706> | 218 | 4 | 0.44634469 | 8.96168376 | 9.84E-04 | 0.47196097 | geneontology_Biological_Process_noRedundant | EMP2;MMP2;NR2F2;PCSK5 |
| 4 | GO:0051348 | negative regulation of transferase activity | <http://amigo.geneontology.org/amigo/term/GO:0051348> | 266 | 4 | 0.54462243 | 7.34453782 | 0.00204524 | 0.69623161 | geneontology_Biological_Process_noRedundant | IGF1R;NR2F2;PDCD4;SOCS3 |
| 4 | GO:0008214 | protein dealkylation | <http://amigo.geneontology.org/amigo/term/GO:0008214> | 33 | 2 | 0.06756594 | 29.600713 | 0.00206464 | 0.69623161 | geneontology_Biological_Process_noRedundant | ARID5B;KDM5C |
| 4 | GO:0001227 | DNA-binding transcription repressor activity, RNA polymerase II-specific | <http://amigo.geneontology.org/amigo/term/GO:0001227> | 267 | 4 | 0.54666988 | 7.31703018 | 0.00207335 | 0.69623161 | geneontology_Molecular_Function_noRedundant | ARID5B;BHLHE40;KDM5C;NFIL3 |
| 5 | GO:0043062 | extracellular structure organization | <http://amigo.geneontology.org/amigo/term/GO:0043062> | 400 | 9 | 0.86715645 | 10.37875 | 1.32E-07 | 4.36E-04 | geneontology_Biological_Process_noRedundant | COL1A1;COL3A1;CST3;DCN;LUM;PLTP;POSTN;SFRP2;SULF1 |
| 5 | GO:0009612 | response to mechanical stimulus | <http://amigo.geneontology.org/amigo/term/GO:0009612> | 207 | 7 | 0.44875346 | 15.5987654 | 2.60E-07 | 4.36E-04 | geneontology_Biological_Process_noRedundant | CHI3L1;COL1A1;COL3A1;DCN;GADD45A;POSTN;TXNIP |
| 5 | GO:0031012 | extracellular matrix | <http://amigo.geneontology.org/amigo/term/GO:0031012> | 496 | 9 | 1.075274 | 8.36995968 | 8.07E-07 | 8.71E-04 | geneontology_Cellular_Component_noRedundant | CHI3L1;COL1A1;COL3A1;DCN;LUM;POSTN;PTN;SFRP2;SULF1 |
| 5 | GO:0005201 | extracellular matrix structural constituent | <http://amigo.geneontology.org/amigo/term/GO:0005201> | 158 | 6 | 0.3425268 | 17.5168776 | 1.04E-06 | 8.71E-04 | geneontology_Molecular_Function_noRedundant | CHI3L1;COL1A1;COL3A1;DCN;LUM;POSTN |
| 5 | GO:0001525 | angiogenesis | <http://amigo.geneontology.org/amigo/term/GO:0001525> | 487 | 8 | 1.05576298 | 7.57745836 | 7.58E-06 | 0.0050929 | geneontology_Biological_Process_noRedundant | CHI3L1;DCN;NRP2;PTN;SAT1;SFRP2;SULF1;WARS |
| 5 | R-HSA-3000178 | ECM proteoglycans | <http://reactome.org/PathwayBrowser/#/R-HSA-3000178> | 76 | 4 | 0.16475973 | 24.2777778 | 2.14E-05 | 0.01195032 | pathway_Reactome | COL1A1;COL3A1;DCN;LUM |
| 5 | GO:0005581 | collagen trimer | <http://amigo.geneontology.org/amigo/term/GO:0005581> | 87 | 4 | 0.18860653 | 21.2081737 | 3.64E-05 | 0.01747254 | geneontology_Cellular_Component_noRedundant | COL1A1;COL3A1;DCN;LUM |
| 5 | GO:1901342 | regulation of vasculature development | <http://amigo.geneontology.org/amigo/term/GO:1901342> | 313 | 6 | 0.67854992 | 8.84238552 | 5.17E-05 | 0.02169556 | geneontology_Biological_Process_noRedundant | CHI3L1;DCN;PTN;SFRP2;SULF1;WARS |
| 5 | GO:0031667 | response to nutrient levels | <http://amigo.geneontology.org/amigo/term/GO:0031667> | 483 | 7 | 1.04709141 | 6.68518519 | 6.77E-05 | 0.02327297 | geneontology_Biological_Process_noRedundant | COL1A1;HMGCL;IFI16;LDHA;POSTN;PTN;SFRP2 |
| 5 | GO:0071496 | cellular response to external stimulus | <http://amigo.geneontology.org/amigo/term/GO:0071496> | 330 | 6 | 0.71540407 | 8.38686869 | 6.93E-05 | 0.02327297 | geneontology_Biological_Process_noRedundant | COL1A1;GADD45A;IFI16;POSTN;PTN;SFRP2 |

**Table S3**

| Primer | Sequence 5' → 3' |
| --- | --- |
| CYP3A4-F | GAAACACAGATCCCCCTGAA |
| CYP3A4-R | CTGGTGTTCTCAGGCACAGA |
| CYP3A7-F | AAGTCTGGGGTATTTATGACT |
| CYP3A7-R | CGCTGGTGAATGTTGGAGAC |
| ALB-F | GCACAGAATCCTTGGTGAACAG |
| ALB-R | ATGGAAGGTGAATGTTTTCAGCA |
| AFP_F | AAATGCGTTTCTCGTTGCTT |
| AFP_R | GCCACAGGCCAATAGTTTGT |
| A1AT_F | ACATTTACCCAAACTGTCCATT |
| A1AT_R | GCTTCAGTCCCTTTCTCGTC |
| OATP1B3-F | TCATTGGCTTTGCACTGGGA |
| OATP1B3-R | AAACCAAGCCACCAAGCTCC |
| NTCP-F | CATGCGCTATGTCATCAAGG |
| NTCP-R | TGATGCTCTTCCCCACATT |
| GRa(NR3C1)-F | TACCCTGCATGTACGACCAA |
| GRa(NR3C1)-R | TCCTTCCCTCTTGACAATGG |
| CYP1A2-F | TGTTCAAGCACAGCAAGAAGG |
| CYP1A2-R | TGCTCCAAAGATGTCATTGAC |
| CYP2A6-F | GAGTCAAAAAGGACACCAAG |
| CYP2A6-R | GCCCCTTCTCATTCAGGAAG |
| CYP2C8-F | TGGCATTACTGACTTCCGTG |
| CYP2C8-R | CCCTTTGGTAACTGCAGTAG |
| CYP2D6-F | GAAGGATGAGGCCGTCTGGG |
| CYP2D6-R | GGAAAGCAAAGACACCATGG |
| CYP2E1-F | GACAGAGACCACCAGCACAA |
| CYP2E1-R | GGTGATGAACCGCTGAATCT |
| CYP7A1-F | GAGAAGGCAAACGGGTGAAC |
| CYP7A1-R | GCACAACACCTTATGGTATGACA |
| ACTB-F | TGGCACCCAGCACAATGAA |
| ACTB-R | CTAAGTCATAGTCCGCCTAGAAGCA |
| GAPDH-F | GCACCGTCAAGGCTGAGAAC |
| GAPDH-R | TGGTGAAGACGCCAGTGGA |
